# Beyond Bactericidal: Plasma Surface Engineering to Defeat Food Matrix‐Conditioning Layers

**DOI:** 10.1111/1541-4337.70663

**Published:** 2026-10-01

**Authors:** Rafael Bianchini Fulindi, Argemiro Soares da Silva Sobrinho, Anderson S. Sant'Ana

**Affiliations:** ^1^ Department of Food Science and Nutrition, Faculty of Food Engineering University of Campinas Campinas Brazil; ^2^ Division of Fundamental Sciences, Department of Physics Aeronautics Institute of Technology (ITA) São José dos Campos Brazil

**Keywords:** biofilm control, cold plasma surface engineering, conditioning films, food contact materials, mechanotransduction, *Salmonella* spp

## Abstract

The persistence of foodborne pathogens on industrial food contact surfaces continues to challenge global food safety despite advances in sanitation technologies. A central limitation of current antimicrobial strategies lies in their validation under simplified laboratory conditions that overlook the physicochemically driven formation of food matrix conditioning films. Upon contact with food residues, organic macromolecules reorganize at the solid‒liquid interface, forming conditioning layers that mask engineered surface functionalities and facilitate microbial attachment. This review examines how such interfacial transformations constrain conventional bactericidal approaches and contribute to sanitization failures in industrial environments. Plasma surface engineering is evaluated as a matrix‐aware strategy capable of tailoring surface energy, hydration behavior, and nanoscale architecture through plasma‐enhanced chemical vapor deposition and magnetron sputtering. These approaches may mitigate organic fouling and modulate bacterial surface sensing under controlled conditions. Particular attention is given to mechanotransduction pathways implicated in early biofilm formation, highlighting how nanoscale surface cues influence c‐di‐GMP signaling and biofilm commitment in both Gram‐negative and Gram‐positive foodborne pathogens, including *Salmonella* spp. and *Listeria monocytogenes*. Significant translational gaps remain, including long‐term durability under repeated cleaning‐in‐place cycles, antimicrobial transport through complex conditioning films, and adaptive tolerance under chronic exposure. This review situates plasma surface engineering within a preventive interfacial design framework, offering an evidence‐based rationale for the development of food contact materials capable of meeting the durability, safety, and regulatory demands of modern processing environments.

## 1 Introduction

Despite decades of advances in chemical sanitation, hygienic design, and regulatory frameworks, foodborne outbreaks associated with pathogens of industrial relevance, particularly *Salmonella* spp. and *Listeria monocytogenes*, remain a leading cause of preventable illness worldwide (Murray et al. 2022). Modern food processing facilities invest billions of dollars annually in cleaning and disinfection programs, yet recurrent contamination events continue to compromise public health and impose substantial economic costs on producers and regulatory agencies alike (Alonso et al. 2023; Mevo et al. 2021). This pattern suggests that recurrent contamination may reflect not only limitations in antimicrobial efficacy but also an incomplete integration of surface physicochemical complexity into current sanitation frameworks (Chowdhury et al. 2025; Katsigiannis et al. 2022).

Most antimicrobial surface technologies are conceived and validated under controlled laboratory conditions, where bacterial adhesion is evaluated using saline solutions or simplified growth media against clean material surfaces (D. C. S. Evans et al. 2023). These assays provide mechanistic insight but do not capture the physicochemical complexity of real processing environments (Giaouris et al. 2014). Industrial food contact surfaces are continuously exposed to organic residues, including proteins, lipids, and carbohydrates originating from raw materials and processing fluids, which accumulate rapidly and transform the interface before microbial attachment occurs (Shi and Zhu 2009). This transformation, driven by physicochemical processes rather than microbial adaptation, likely contributes to the loss of functionality observed when surfaces that exhibit strong antimicrobial or antifouling properties in vitro are exposed to industrial environments (Sadekuzzaman et al. 2015; Afonso et al. 2024).

The formation of this transformed interface is governed by fundamental thermodynamic principles: when solid surfaces come into contact with food fluids, organic macromolecules spontaneously adsorb onto the interface (Bouazizi et al. 2022). This process is driven by the gain in system entropy associated with the displacement of structured interfacial water molecules (Hage et al. 2022). Proteins and other macromolecules replace surface‐bound water within seconds, forming a molecular conditioning film (van den Berg et al. 2024). Once established, this organic layer masks the original surface chemistry and topography, redefining the physicochemical cues perceived by approaching bacterial cells and altering both adhesion dynamics and antimicrobial performance (Mu et al. 2023; D. Liu et al. 2022). Bacteria do not attach to the engineered material itself but rather to this biologically active organic layer, which provides anchoring sites, neutralizes surface charge, and attenuates antimicrobial activity (Sacher and Yelon 2021), a mechanism that helps explain why many bactericidal coatings and chemically active surfaces perform well in vitro but fail under industrial conditions (Y. Zhao et al. 2023).

Current food safety strategies are divided along two parallel tracks, neither of which explicitly accounts for the conditioning film problem. Industrial sanitation relies heavily on wet chemical disinfectants, which are readily neutralized by organic matter and exhibit limited penetration into established biofilms (Mevo et al. 2021). Repeated applications of these agents can also promote sublethal stress responses, potentially favoring biofilm formation or the emergence of viable but non‐culturable (VBNC) states (Arvaniti et al. 2021). In parallel, academic research has produced advanced antifouling polymers and bactericidal nanomaterials that perform well under controlled conditions, but often lack mechanical robustness, long‐term stability, or scalability for food processing applications (Akdoğan and Şirin 2021). In both cases, the material interface under organic challenge remains the unaddressed variable (Hage et al. 2022; Mu et al. 2023). In this context, plasma surface engineering represents a promising strategy to address these interfacial challenges by enabling controlled modification of surface chemistry and energy through ionized gas processes (Dufour 2023). Unlike conventional wet chemical treatments, plasma processes operate in dry, solvent‐free environments and can be applied uniformly to complex industrial geometries (Puligundla and Mok 2017). These characteristics make plasma technologies attractive candidates for food processing applications subjected to repeated mechanical stress and aggressive cleaning cycles, although long‐term industrial validation remains limited (Y. M. Zhao, Patange, et al. 2020).

Beyond their technological advantages, plasma‐based approaches have the potential to enable the simultaneous manipulation of surface physics and the physicochemical cues relevant to bacterial surface sensing (Chiappim et al. 2021). By manipulating how surfaces retain or repel interfacial water, together with surface energy and nanoscale features, plasma‐engineered interfaces may modulate the physicochemical cues available to approach bacterial cells (Y. Cheng et al. 2023). Such cues have been associated with surface sensing, mechanotransduction, and the upstream activation of biofilm regulatory pathways in Salmonella and related pathogens, although direct causal links in food processing contexts remain to be established (Zheng et al. 2021). Rather than relying solely on contact killing, plasma surface engineering offers the possibility of designing interfaces that resist matrix interference and suppress the conditions required for stable bacterial adhesion (Özdemir et al. 2023).

Recent reviews have broadly addressed cold plasma technology in the context of food processing, covering microbial inactivation, decontamination of fresh produce, food functionality enhancement, and packaging material modification (Keewan et al. 2025; Sasikumar et al. 2025; Shill and Sit 2025). While these contributions consolidate the evidence base for plasma as a nonthermal treatment, the present review differs in scope and conceptual framing. Rather than evaluating plasma as a direct decontamination agent applied to foods or packaging, this work examines plasma surface engineering as a permanent interfacial modification strategy for food contact materials. We propose that food matrix conditioning film formation represents a central physicochemical constraint governing the long‐term failure of antimicrobial surfaces under industrial conditions. By connecting the thermodynamics of conditioning film assembly, the engineering parameters of plasma‐deposited coatings, and the molecular mechanisms of bacterial mechanotransduction within a single explanatory framework, this review provides an integrative mechanistic perspective for interpreting why many antimicrobial strategies underperform outside controlled laboratory settings and identifies interfacial characteristics associated with improved performance under food processing conditions. This framework is particularly timely given the sustained increase in research activity focused on cold plasma surface modification over the past decade, with annual publication output rising approximately sixfold between 2015 and 2025, based on PubMed‐indexed records of studies on cold plasma surface engineering, functionalization, and bacterial biofilm control.

## 2 Food Matrix Interference as an Interfacial Phenomenon

### 2.1 Inevitability of Conditioning Film Formation Under Industrial Conditions

The persistent gap between laboratory antimicrobial performance and industrial efficacy reflects, in part, the thermodynamically driven formation of conditioning films at food contact surfaces under real processing conditions (Sacher and Yelon 2021). Under controlled laboratory conditions, microbial adhesion is commonly examined as a direct interaction between a clean material surface and a bacterial cell (Azeredo et al. 2017). This reductionist framework enables detailed mechanistic analysis but does not capture the physicochemical complexity of industrial food processing systems (Y. M. Zhao, Patange, et al. 2020). In practice, food contact materials, including AISI 304 and 316 stainless steel, are never encountered in a pristine state (Carpentier and Cerf 2011).

Immediately upon exposure to food fluids, these surfaces undergo rapid modification by organic constituents originating from raw materials, processing operations, and residual product deposits (Chowdhury et al. 2025). This process, commonly referred to as organic fouling, is a ubiquitous feature of food processing environments and represents not a random accumulation of debris but a structured thermodynamically driven phenomenon (Barberi and Spriano 2021). Proteins, lipids, and polysaccharides present in food matrices adsorb onto solid surfaces within seconds to minutes, forming a molecular conditioning film that substantially alters the physicochemical character of the solid‒liquid interface (Hage et al. 2022).

Once established, the conditioning film obscures native surface chemistry, charge distribution, and nanotopography, such that bacterial adhesion, survival, and early biofilm development are mediated by this organic layer rather than by the engineered substrate itself (Pinto et al. 2023; Whitehead et al. 2019; Chowdhury et al. 2025).

Empirical observations from dairy, meat, and fresh produce processing environments confirm that organic residues persist on stainless steel surfaces despite aggressive cleaning protocols, with irreversible fouling emerging within days in filtration systems, heat exchangers, and conveyor assemblies (Chowdhury et al. 2025; Katsigiannis et al. 2022; Basiri et al. 2023).

### 2.2 Thermodynamic Drivers of Conditioning Film Formation

The spontaneous formation of conditioning films is governed by the thermodynamics of macromolecular adsorption at solid‒liquid interfaces. Protein adsorption at solid‒liquid interfaces is described by the Gibbs free energy relationship:

ΔG=ΔH−TΔS

where Δ*G* represents the change in free energy (the potential for the process to occur), Δ*H* the change in enthalpy (heat and bonding forces), and *T*Δ*S* the product of absolute temperature and the change in entropy (the degree of system disorder). In food processing systems, even when the direct chemical attraction between the protein and the surface (Δ*H*) is weak or unfavorable, the process becomes spontaneous due to the substantial increase in system entropy. This entropic gain is primarily associated with the displacement of structured interfacial water molecules that form the highly ordered hydration layer at the solid‒liquid interface (Barberi and Spriano 2021).

On submerged solid surfaces, water molecules organize into highly ordered hydration layers stabilized by hydrogen bonding and electrostatic interactions. When macromolecules such as proteins approach the interface, these structured water molecules are expelled into the bulk phase (Tandon et al. 2008). The release of constrained water molecules results in a significant entropy gain, rendering adsorption energetically favorable. This dehydration‐driven process occurs rapidly and is largely irreversible under industrial conditions due to protein conformational rearrangements and multiple binding interactions with the surface (Mu et al. 2023).

The displacement of interfacial water often induces partial protein unfolding, exposing hydrophobic domains that enhance surface affinity and promote irreversible anchoring of the conditioning layer (Sacher and Yelon 2021). As a result, materials incapable of sustaining a tightly bound hydration barrier are thermodynamically predisposed to organic fouling, regardless of their initial surface smoothness or cleanliness (Chowdhury et al. 2025).

This thermodynamic framework explains the observed loss of engineered surface properties in conventional food contact materials upon exposure to complex food matrices (Whitehead et al. 2019) and underscores the relevance of water structure and entropy‐driven adsorption as design parameters for antifouling strategies (Mu et al. 2023).

### 2.3 Thermodynamic Constraints on Passive Surface Strategies: The Vroman Effect

Attempts to mitigate organic fouling through simple surface activation, such as increased hydrophilicity or elevated surface energy, frequently fail under industrial conditions. High‐energy surfaces can paradoxically accelerate protein adsorption due to the Vroman effect, a dynamic adsorption process in which abundant, low‐affinity proteins initially bind to the surface and are progressively displaced by lower abundance, higher affinity proteins (Afonso et al. 2024).

In complex food matrices, this continuous exchange of adsorbed macromolecules is unavoidable and leads to the rapid formation of heterogeneous conditioning films that mask both surface chemistry and nanotopography within minutes (Mu et al. 2023). Therefore, purely passive physical modifications lose antifouling functionality almost immediately following exposure to food residues, despite performing well under clean laboratory conditions (van den Berg et al. 2024).

The magnitude of these Vroman‐driven dynamics varies substantially across food matrices, with direct consequences for conditioning film composition and antimicrobial surface functionality. In protein‐rich dairy environments, caseins and whey proteins adsorb via entropy‐driven displacement of interfacial water, forming relatively ordered proteinaceous films that attenuate antimicrobial efficacy primarily through chemical scavenging of reactive oxygen and nitrogen species (RONS) (Mu et al. 2023; Katsigiannis et al. 2022). In contrast, meat and poultry exudates introduce myofibrillar proteins alongside lipid fractions, the latter of which progressively enrich the interface through high‐affinity hydrophobic interactions. This lipid co‐adsorption disrupts the structured hydration layer more severely, generating heterogeneous films with hydrophobic domains that impose both chemical scavenging and physical diffusion barriers against biocidal ions and reactive species (Hadinoto et al. 2023; Katsigiannis et al. 2022). The distinction is not merely quantitative: dairy matrices compromise antimicrobial functionality primarily through molecular interference, whereas meat‐derived conditioning films combine molecular and physical occlusion, imposing compounded challenges for surfaces intended to perform across multiple processing contexts.

Passive strategies that rely exclusively on surface energy modulation or static topographical features are inherently vulnerable to these protein exchange dynamics (Afonso et al. 2024). Once obscured by the conditioning layer, such surfaces no longer present the intended physicochemical cues to bacterial cells, suggesting that passive surface energy modulation alone is insufficient to sustain antimicrobial functionality under organic‐rich conditions (Y. Cheng et al. 2023).

Plasma‐engineered coatings capable of stabilizing and delivering biocidal ions, such as Zn^2+^, represent one potential approach to extending antimicrobial activity beyond the limitations of purely passive strategies (Akdoğan and Şirin 2021). Unlike static surface features, diffusible antimicrobial species may penetrate the conditioning film and exert antimicrobial pressure at the bacteria–interface junction, potentially preserving functionality even when the underlying surface chemistry is partially masked by organic fouling (DeFlorio et al. 2021).

These matrix‐dependent constraints are documented across a broad body of primary experimental studies, summarized in Table 1, which compiles reported organic loads, exposure conditions, adsorption mechanisms, and quantitative outcomes for dairy, red meat, poultry, and fresh produce matrices. Across all four matrix classes, conditioning film formation consistently precedes and modulates microbial attachment, yet the dominant adsorption mechanism and the resulting interfacial consequence differ systematically between protein‐dominated and lipid‐enriched systems.

**TABLE 1:** Matrix‐dependent mechanisms of conditioning film formation on food contact surfaces, compiled from primary experimental studies.

Matrix	Organic load	Exposure conditions	Adsorption mechanism	Interfacial consequence	Quantitative result	References
Dairy	Caseins and whey proteins	SS 304, 4°C–25°C, 1–24 h, pH 6.7	Entropy‐driven water displacement and hydrophobic interaction	Surface charge and energy masking by protein layer	Biocide efficacy limited to ∼1.0 log CFU/cm^2^ reduction	Alonso et al. (2023)
Dairy	Fat globules and proteins (β‐lactoglobulin, casein)	SS 304, 20°C, 1 h, pH 6.6	Competitive protein–lipid adsorption (Vroman effect)	Increased hydrophobicity and contact angle change	1.8× increase in *Staphylococcus aureus* adhesion (10^6^ CFU/cm^2^)	Hamadi et al. (2014)
Dairy	Whey proteins and caseins (1–10 mg/mL)	SS 304 and Buna‐N rubber, 4°C–21°C, 20 min–2 h	Irreversible hydrophobic binding to active sites	Passivation of native sites; secondary receptor layer	Adhesion drop 10^5^ → 10^3^ CFU/cm^2^, but protected against chlorine	Helke et al. (1993)
Dairy	Pure β‐lactoglobulin and milk proteins (0.1%–2.0%)	Metals (20–70 mN/m), 20°C–75°C, pH 6.6	Surface‐induced protein unfolding	Irreversible surface free energy shift	Film thickness 5–35 nm; contact angle 45° → > 75°	McGuire and Swartzel (1989)
Dairy	Heat‐denatured whey proteins and calcium phosphate	SS 316L, 65°C–72°C, 30 min–4 h	Co‐precipitation via Ca‐bridges	Dense, rough mineral‐protein fouling	Surface roughness (Ra) 0.2 → 1.4 µm	Flint et al. (2000)
Dairy	Raw milk (whole/pasteurized)	SS 316L (control) vs. Thermolon sol–gel modified coupons, 72°C, 6 h	Sol–gel surface passivation reducing protein–lipid co‐deposition	Lower surface energy limits fouling layer buildup	Fouling weight 19.21 → 0.37 mg/cm^2^ on modified SS	D. Z. Liu et al. (2017)
Dairy	Whey protein concentrate (10 g/L)	Plasma‐treated silane SS 2B, 85°C, 2 h	Thermal desaturation and van der Waals binding	Masking of silanized high‐energy coating	QCM‐D: 65% reduction in deposit mass	Zouaghi et al. (2018)
Dairy	Reconstituted whey proteins and minerals	Nanotextured/fluorinated SS 316L, 80°C, 90 min	Cassie‐Baxter air‐trapping	Localized nanocavity filling by protein aggregates	Contact angle retained 130°; 80% drop in adhered *Listeria*/*Pseudomonas*	Zouaghi et al. (2019)
Dairy	Whole milk proteins and lipids	SS and glass, 4°C–20°C, 2–24 h	Physical adsorption and rearrangement	Viscoelastic protective conditioning film	Ultrasonic removal of *Escherichia coli* biofilm reduced 40%	Oulahal‐Lagsir et al. (2003)
Red meat	Beef exudate (myoglobin, myosin, sarcoplasmic proteins)	SS 304, PS, rubber, 4°C–25°C, pH 5.8, 15 min–24 h	Rapid interfacial water displacement (Vroman)	Complete surface charge masking	Increased shear stress needed for *Pseudomonas fluorescens* detachment	Piette and Idziak (1992)
Red meat	Beef/pork exudates (2–5 mg/mL)	SS 304 2B, 10°C, pH 5.6–6.2, 1–4 h	Irreversible hydrophobic interaction	Viscoelastic layer as bacterial anchor	Biofilm removal efficacy 99.9% → < 85%	Oulahal et al. (2007)
Red meat	Meat plant residues (proteins, lipids, myoglobin)	SS and PVC/PE, 12°C, 48 h continuous	Co‐adsorption of lipids and proteins	Physical diffusion barrier	Protected *Listeria monocytogenes* against QAC/PAA by up to 3.5 logs	Fagerlund et al. (2017)
Red meat	Pork exudate (proteins and lipids)	SS 304 and PVC, 15°C, 2–12 h	Multiphase adsorption, lipid‐preferential	Hydrophilic → moderately hydrophobic shift	Fourfold increase in *L. monocytogenes*/*Salmonella* retention	Ripolles‐Avila et al. (2022)
Red meat	Pork skin, beef and lamb carcass tissue (wax‐embedded)	4°C–21°C, 30 min	Physical entrapment and hydrophobic forces at native tissue surface	Irregular tissue topography creates micro‐cavities favoring retention	Primary bacterial retention increased up to 2.5 logs CFU/cm^2^	Butler et al. (1979)^a^
Poultry	Chicken juice (skin lipids, proteins, 10%–100%)	SS 304 and PS, 4°C–37°C, 1–24 h	Rapid hydrophobic lipid binding	Adhesive lipid–protein layer in micro‐scratches	*Campylobacter jejuni*/*Salmonella* adhesion > 100‐fold (2 logs CFU/cm^2^)	Brown et al. (2014)
Poultry	Chicken skin exudate (fat, sarcoplasmic proteins)	SS, 50°C–90°C, seconds–min	Thermal lipid spreading and protein fixation	Continuous viscous hydrophobic film	Steam kill rate reduced by 3.0 logs	Kondjoyan and Portanguen (2008)
Poultry	Meat exudate + *E. coli*	SS/Ti‐coated SS, linear topography, wipe cleaning	Topography‐dependent retention	Regular/Ti‐coated surfaces easier to clean	No diff. in bacteria after 10 cleans; exudate removed easier along linear features	A. Evans et al. (2021)
Poultry	Chicken juice biofilm matrix (single vs. dual‐species)	SS coupons, 25°C, pH 7.0, 6‐day biofilm	Glycoconjugate‐enriched matrix in dual‐species biofilm	Matrix shielding reduces chlorine penetration	Dual‐species biofilm: ∼1 log CFU/cm^2^ lower chlorine reduction vs. single‐species	Pang and Yuk (2018)
Poultry	Poultry house/slaughter residues	SS and PS, 20°C–25°C, up to 48 h	Multiphase protein–lipid adsorption	Surface energy modification, anchor sites	Significant increase in *Salmonella enterica* biomass	Paz‐Méndez et al. (2017)
Poultry	Chicken breast/thigh exudate	Chicken drumstick (skin‐on) and breast meat (skinless), attachment periods 0.5–210 min	Time‐dependent progressive protein adsorption	Protective diffusion barrier layer	Sanitizer kill log reduced up to 2.2 logs for *Listeria*/*Salmonella*	İlhak et al. (2018)
Poultry	Raw chicken breast micro‐residue	SS SUS304, 20°C–25°C, ambient drying	Physical adsorption and desiccation	Dry conditioning film, ATP/AMP retained	A3 bioluminescence signal exceeded the 200 RLU hygiene benchmark before detergent washing	Saito et al. (2020)
Produce	Lettuce/endive juice (pectin, cellulose, plant protein)	SS 304, glass, PS, 10°C–20°C, pH 6.2, 2 h	Hydrogen bonding, polysaccharide‐OH to metal oxide	Hydrated gel‐like hydrophilic layer	Desiccation survival of *Salmonella*/*E. coli* O157:H7 +3 logs	Schlisselberg and Yaron (2013)
Produce	Hydroponic and soil‐grown lettuce leaf extract	SS coupons, 4°C and 10°C	Nutrient‐rich leaf extract supports sessile growth	Biofilm establishment enhanced by leaf exudate presence	Biofilm increased 3 → 6.4–7.2 log CFU/cm^2^ (10°C); 3 → 4.3–4.8 log CFU/cm^2^ (4°C)	Kyere et al. (2020)
Produce	Plant sap/citrus juice extract (pectin, sugars, acids)	SS and silicon, 25°C, pH 3.5–5.5, AFM	Polysaccharide self‐assembly, van der Waals/H‐bonds	Altered nanoroughness, Young's modulus gradient	3× increase in single‐cell adhesion force (AFM)	Lorite et al. (2011)

*Note*: Entries report the organic load, experimental exposure conditions, proposed adsorption mechanism, resulting interfacial consequence, and quantitative outcome for dairy, red meat, poultry, and fresh produce matrices. Reported log reductions refer to the antimicrobial or cleaning treatment specified in each source study and are not directly comparable across studies, given differences in substrate, temperature, contact time, and target microorganism. Reported log reductions refer to the antimicrobial or cleaning treatment specified in each source study and are not directly comparable across studies.

Abbreviations: AFM, atomic force microscopy; AMP, adenosine monophosphate; ATP, adenosine triphosphate; CFU, colony‐forming units; HDPE, high‐density polyethylene; OCP, open circuit potential; PAA, peracetic acid; PE, polyethylene; PP, polypropylene; PS, polystyrene; PVC, polyvinyl chloride; QAC, quaternary ammonium compounds; QCM‐D, quartz crystal microbalance with dissipation monitoring; Ra, arithmetic mean surface roughness; RLU, relative light units; SS, stainless steel.

^a^ Butler et al. (1979) evaluated bacterial attachment on native pork skin and beef/lamb carcass tissue rather than food contact equipment surfaces; included as a biological model of irregular topography‐driven retention.

### 2.4 Implications for Antimicrobial Surface Engineering

The interfacial transformations described in this section collectively constrain antimicrobial surface performance through mechanisms that are largely independent of biocidal potency (Z. S. Xu et al. 2023).

These interfacial constraints define the boundary conditions for viable surface engineering approaches: candidate strategies must either resist conditioning film formation or retain antimicrobial functionality beneath it, even when surface chemistry and energy are rapidly altered by food residues (Hua and Zhu 2024a).

This sequence of interfacial transformations, from a transient hydrated state to a biologically active conditioning film, dictates microbial behavior at food contact surfaces and constrains antimicrobial performance, as summarized in Figure 1.

**FIGURE 1:**
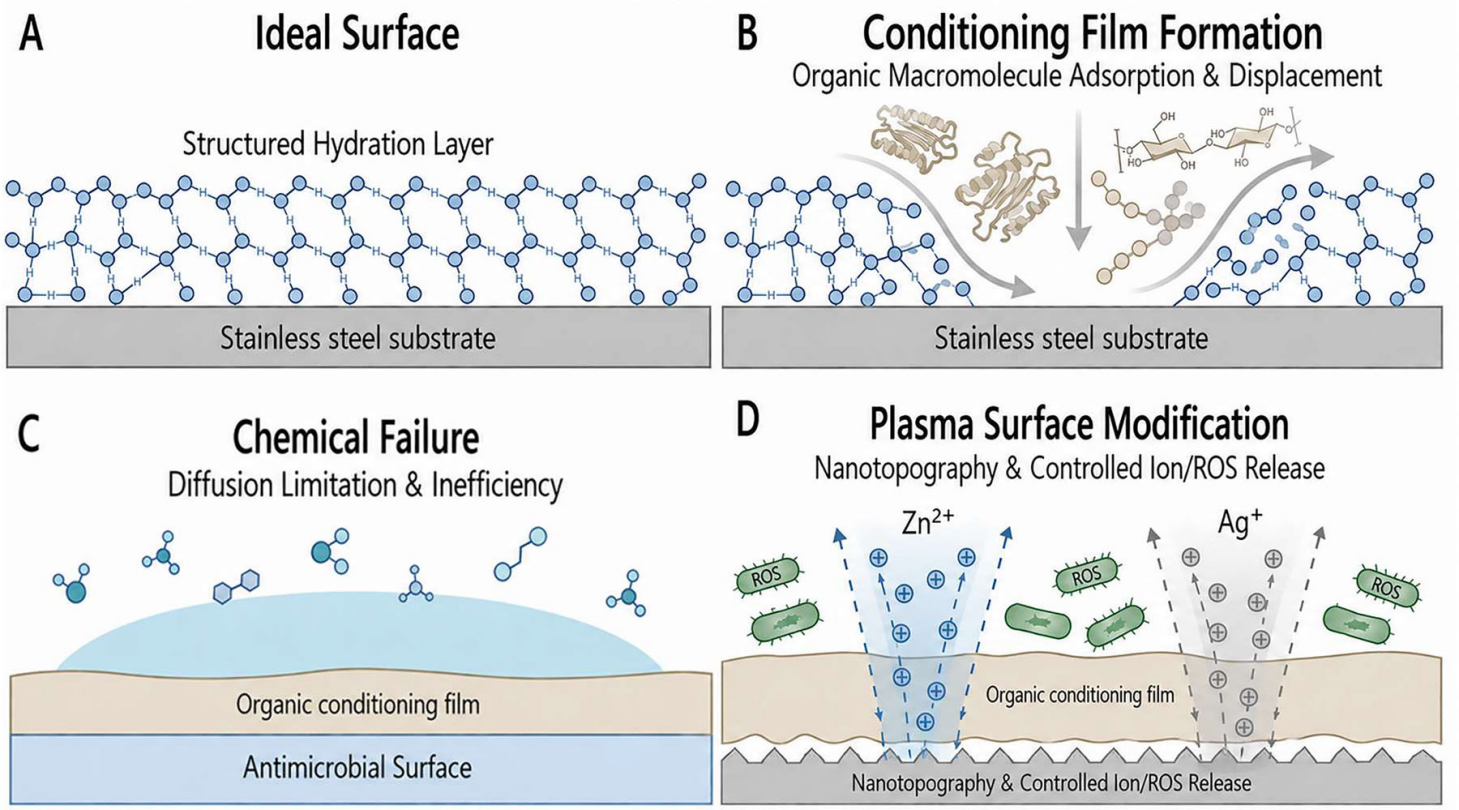
Dynamic evolution of food contact surfaces after cleaning and its implications for microbial adhesion and persistence. Panel A represents the immediate post‐cleaning state of stainless steel, characterized by a structured hydration layer that is transient and thermodynamically unstable under real processing conditions. Panel B illustrates the rapid adsorption of proteins, lipids, and polysaccharides derived from food matrices, resulting in the formation of a conditioning film that obscures surface chemistry and reshapes early bacterial sensing and attachment processes. Panel C depicts the functional failure of conventional antimicrobial strategies under fouling conditions, where the conditioning layer facilitates stable adhesion, promotes extracellular matrix development, and attenuates biocidal efficacy. Panel D shows plasma‐engineered surfaces operating under fouling conditions, in which controlled ion diffusion may sustain antimicrobial functionality beneath the conditioning film (Akdoğan and Şirin 2021; DeFlorio et al. 2021). Figure created by the authors based on the mechanisms discussed in Sections 2.1–2.4 of this review.

Collectively, these thermodynamic constraints demonstrate that evaluating antimicrobial surfaces exclusively under clean laboratory conditions creates a fundamental disconnect from industrial reality. The rapid masking of surface free energy and topography by food matrix constituents (as outlined in Table 1) establishes that passive antifouling designs are inherently self‐limiting in organic‐rich environments. To move beyond this limitation, the engineering of next‐generation food contact materials must pivot from passive static surface modifications toward active, multitiered architectures. Such systems must integrate sustained subsurface ion release (e.g., controlled Zn^2+^ diffusion) or dynamic interfacial energy tuning capable of penetrating or disrupting the conditioning film, thereby maintaining biocidal pressure directly at the transient bacteria–substrate interface.

## 3 Plasma Surface Engineering: Mechanisms and Functional Capabilities

Conventional wet chemical sanitation remains the dominant industrial approach due to its low initial cost and regulatory familiarity (Torres et al. 2025). However, these methods suffer from intrinsic limitations, including rapid neutralization by organic matter, lack of residual activity, environmental burden, and limited effectiveness in complex industrial geometries (Galié et al. 2018).

Advanced antifouling materials, particularly zwitterionic and superhydrophilic coatings, demonstrate exceptional resistance to protein adsorption under controlled laboratory conditions (S. Liu et al. 2022). Despite their performance in clean systems, these materials often exhibit rapid functional degradation in real food processing environments due to mechanical abrasion, repeated cleaning‐in‐place (CIP) cycles, and heterogeneous organic loads characteristic of dairy, meat, and fresh produce processing lines (Y. Cheng et al. 2023; Chowdhury et al. 2025). This discrepancy illustrates the translational gap between laboratory‐optimized surface chemistries and industrial applicability (Akdoğan and Şirin 2021).

This gap is particularly evident when considering potent antimicrobial agents synthesized via wet chemistry. For instance, zinc‐based nanoparticles (ZnO and ZnS) have demonstrated exceptional efficacy in disrupting established biofilms and protecting biological matrices such as collagen (Fulindi et al. 2023, 2026). Similarly, the precise morphological control of silver nanoparticles (AgNPs) has been shown to correlate strongly with robust antimicrobial responses (J. A. O. Santos et al. 2024). However, while these nanostructures are highly effective in controlled or biomedical settings, they often lack the interfacial adhesion required to survive the continuous mechanical abrasion and aggressive CIP cycles of food processing lines.

Plasma surface engineering represents one approach to addressing this translational challenge (Dufour 2023). Rather than replacing zwitterionic or antifouling concepts, plasma‐based technologies enable their stabilization, anchoring, and functional integration onto industrial substrates (Sainz‐García et al. 2022). Plasma surface engineering should not be regarded as a sanitization method in isolation, but as an interfacial engineering platform whose primary role is to stabilize surface chemistries and preserve antimicrobial performance under realistic industrial conditions characterized by continuous organic loading, complementing rather than replacing established chemical or physical sanitation procedures (Gilmore et al. 2018; Katsigiannis et al. 2022).

Plasma processes employ ionized gases capable of inducing controlled bond scission, generating reactive species, and promoting targeted surface functionalization (Ghezzi et al. 2023). These modifications occur in dry, solvent‐free environments and can be applied uniformly to complex industrial geometries such as conveyor belts, valves, heat exchangers, and stainless steel tanks (Nikiforov et al. 2022). This combination of precision and robustness addresses the demands of food processing systems exposed to repeated mechanical stress and aggressive CIP regimes (Hegemann et al. 2022).

From a functional perspective, plasma surface engineering enables three complementary interfacial control mechanisms that directly address food matrix interference:

- modulation of surface energy (the energetic tendency of a surface to interact with water and organic molecules) and hydration behavior to delay organic adsorption,
- immobilization or encapsulation of antimicrobial agents with diffusion‐controlled release, and
- enhancement of interfacial adhesion between functional coatings and industrial substrates to ensure long‐term durability.

### 3.1 Sputtering: Controlled Metallic Reservoirs

Magnetron sputtering is a physical vapor deposition technique that enables the formation of ultrathin, compositionally homogeneous metallic coatings with nanometric precision. In this process, energetic ions bombard a metallic target, ejecting atoms that subsequently condense onto the substrate surface (Bazaka et al. 2015). For food contact materials, sputtered films typically range from 10 to 500 nm in thickness, enabling precise control over coating composition, surface roughness, and antimicrobial ion release kinetics (Rocha‐Cuervo et al. 2023).

Plasma‐polymerized silver‐containing films exhibit a two‐phase release profile: an initial burst during primary hydration, followed by sustained basal‐level elution over extended contact periods (Körner et al. 2010; Lischer et al. 2011). In Ag‐containing plasma polymer matrices, this kinetic profile maintains inhibitory concentrations at the bacterial interface without the rapid functional depletion characteristic of chemically impregnated coatings, while remaining below acute cytotoxicity thresholds (Lischer et al. 2011). Sputtered Ag/a‐C:H nanocomposite films extend this principle by embedding AgNPs within a hard carbon matrix, confining ion diffusion to the near‐surface zone and coupling sustained antimicrobial activity with the mechanical protection characteristic of diamond‐like carbon (DLC) architectures (Vaidulych et al. 2017; Hanuš et al. 2016). From an industrial perspective, sputtered coatings exhibit strong interfacial adhesion to stainless steel substrates, significantly reducing the risk of delamination during high‐pressure cleaning, thermal cycling, and mechanical abrasion (Juma et al. 2024). These attributes render magnetron sputtering especially suitable for high‐value food processing components with extended operational lifetimes (Sainz‐García et al. 2022).

Beyond conventional DC magnetron sputtering, high‐power impulse magnetron sputtering (HiPIMS) improves film density and interfacial adhesion relevant to food contact applications (Sarakinos et al. 2010). By delivering high‐power pulses at low duty cycles, HiPIMS achieves substantially higher ionization of the sputtered flux compared with DC sputtering, enabling film growth under intense ion bombardment (Bohlmark et al. 2006). This compacts the film microstructure, eliminating the columnar voids characteristic of standard sputtering that represent potential infiltration pathways for cleaning agents under repeated CIP exposure (Sarakinos et al. 2010). Co‐sputtering approaches, such as Ti–Cu thin films deposited by reactive magnetron sputtering, illustrate the compositional flexibility of this platform, yielding films with nanometric surface roughness and synergistic antibacterial activity derived from modified surface energy and electrochemical ion release at the contact interface (Mahmoudi‐Qashqay et al. 2023).

### 3.2 Plasma‐Enhanced Chemical Vapor Deposition: Hybrid Interfaces for Hydration and Controlled Release

Plasma‐enhanced chemical vapor deposition (PECVD), a low‐temperature process in which gaseous precursors are fragmented within the plasma and polymerized directly on the substrate surface, expands the surface design space beyond metallic systems by enabling the fabrication of hybrid organic–inorganic interfacial architectures (Nikiforov et al. 2022). Operating at relatively low temperatures, PECVD is compatible with both metallic and polymeric food contact materials (Laux et al. 2025). During deposition, volatile precursors are fragmented within the plasma and recombine on the substrate surface, forming highly cross‐linked polymeric networks (Gosar et al. 2020).

In the context of food matrix interference, the primary advantage of PECVD lies in its ability to encapsulate antimicrobial agents within a highly cross‐linked protective matrix (Mohammad and Ahmad 2024). This architecture limits immediate neutralization by proteins, lipids, and polysaccharides present in food residues (Palumbo et al. 2020). Instead, antimicrobial activity proceeds via diffusion‐controlled release, allowing functional persistence beneath conditioning films commonly encountered in meat and fresh produce processing environments (Kuzminova et al. 2016).

PECVD also enables fine‐tuning of surface chemistry to stabilize interfacial hydration layers. By incorporating polar or zwitterionic functionalities, plasma‐polymerized coatings increase the energetic penalty associated with water displacement and protein adsorption (Q. Li et al. 2022). While hydration‐mediated antifouling alone is insufficient under industrial conditions, its integration with controlled antimicrobial release may yield a more durable interfacial strategy under organic‐rich conditions (Yu et al. 2015).

Within this framework, PECVD provides the mechanical anchoring required for surface chemistries that demonstrate antifouling performance under controlled conditions to be functionally integrated into industrial substrates (Katsigiannis et al. 2022).

#### 3.2.1 DLC and a‐C:H Architectures

Among PECVD‐derived functional architectures, DLC and hydrogenated amorphous carbon (a‐C:H) films represent the state of the art for food contact applications requiring simultaneous hardness, chemical inertness, and tunable surface energy (Marciano et al. 2009). Deposition of a‐C:H on polymeric substrates via plasma‐assisted PECVD produces graded interfacial layers that ensure structural adhesion of the carbonaceous film under mechanical stress, forming a wear‐resistant barrier compatible with repeated cleaning cycles (Schlebrowski et al. 2021). Incorporation of AgNPs into a‐C:H matrices yields Ag/a‐C:H nanocomposite coatings with surface‐enriched silver distribution, enabling localized antimicrobial ion release while preserving the tribological performance of the carbon matrix (Hanuš et al. 2016; Vaidulych et al. 2017). Flexible DLC films deposited from camphor precursors have demonstrated effective inhibition of *Candida albicans* biofilm formation on polyurethane substrates under mechanical deformation (T. B. Santos et al. 2017), illustrating the potential of carbon‐based PECVD architectures for antimicrobial surface design on flexible food processing components. Wettability control via PECVD has also been demonstrated using lauryl methacrylate‐derived coatings, which can achieve stable superhydrophobic states with water contact angles exceeding 150°, maintained under physical stress through hierarchical roughness anchored by covalent plasma bonding (Xu et al. 2019).

### 3.3 Dielectric Barrier Discharge: Atmospheric Activation and Interfacial Preparation

While sputtering and PECVD offer exceptional nanoscale control, their reliance on vacuum environments can limit throughput for large‐scale components (Y. Liu et al. 2025). Dielectric barrier discharge (DBD) plasma addresses the scalability limitations of vacuum‐based plasma technologies by operating at atmospheric pressure, enabling direct integration into continuous industrial processing lines (Özdemir et al. 2023).

DBD is not a standalone antimicrobial strategy; its primary role is surface activation and interfacial preparation (Ma et al. 2021). Exposure to DBD generates RONS that remove weakly bound organic contaminants while increasing surface free energy and chemical reactivity.

This activation significantly enhances the adhesion of subsequently applied functional coatings, whether metallic or polymeric (Y. Liu et al. 2025). In industrial workflows, DBD can be integrated as a pretreatment step before sputtering or PECVD, ensuring robust interfacial anchoring and long‐term coating stability (Yu et al. 2015).

### 3.4 Comparative Functional Roles of Plasma Technologies

The modular nature of plasma surface engineering allows distinct technologies to be combined into integrated interfacial strategies rather than applied as isolated surface treatments. Their complementary roles in food contact surface design are summarized in Table 2, with the underlying deposition mechanisms illustrated schematically in Figure 2.

**TABLE 2:** Comparative overview of primary studies on plasma deposition technologies (magnetron sputtering, PECVD, and DBD) applied to food‐contact and food‐packaging surfaces.

Technology	Substrate tested	Quantified outcome	References
Sputtering	Stainless steel (heat exchanger)	Reduced milk fouling; deposits more easily removed on lower electron‐donor energy surfaces	Rosmaninho, Santos, et al. (2007)
Sputtering	Extruded PLA film	Strong antibacterial activity against *Escherichia coli*; low release into saline solution	Valerini et al. (2018)
Sputtering	Stainless steel 316L	*Staphylococcus aureus* adhesion reduced from ∼1.8 × 10^6^ to ∼1.4 × 10^6^ CFU/cm^2^ with increasing Si content in DLC	Q. Zhao et al. (2007)
Sputtering	PLA‐PCL films	No antimicrobial activity observed at 1‐min sputtering time	Rybalchenko et al. (2024)
Sputtering	PLA film	UV‐B transmittance reduced ∼95%; > 5 log CFU/cm^2^ inhibition (*E. coli*, 24 h); O_2_ permeation reduced 99.9%	Pedroni et al. (2021)
Sputtering	PEF film	Antibacterial effect and limited adhesion against ∼10^5^ CFU/mL *E. coli*	C. Zhu et al. (2023)
Sputtering	Stainless steel D‐9	*Pseudomonas aeruginosa* load reduced from ∼4 × 10^6^ to 1.2 × 10^2^ CFU on Cu‐doped film (10.46 at% Cu)	Elangovan et al. (2022)
Sputtering	Stainless steel	99.9% antimicrobial inhibition (*E. coli*, *S. aureus*, *Penicillium funiculosum*); safe elution, mean 27.2 µg/L	Shim et al. (2017)
PECVD	PLA film	Highest barrier properties (lowest migration) at 5‐min treatment	Mattioli et al. (2013)
PECVD	Stainless steel	Antifungal activity on sessile cells via controlled Ag^+^ release	Saulou et al. (2009)
PECVD	Stainless steel	Decreased metal content in low‐silver coating (7.5%) after immersion in 0.15 M NaCl	Zanna et al. (2010)
PECVD	Stainless steel (DLC‐coated)	Fouling rate reduced to 1.71 × 10^−4^ m^2^K/W h vs. 2.0 on bare steel	Boxler et al. (2013)
PECVD	PLA film	DEDB plasticizer migration reduced 23.75%–36.90%; O_2_ permeability reduced 48.45%	Y. Zhao, Huang, et al. (2020)
PECVD	UHMWPE	Bacterial reduction: 4.8 log (*E. coli*), 2.1 log (*S. aureus*)	Zabihzadeh Khajavi et al. (2024)
PECVD	PLA film (SiO*_x_*)	Migration reduced: TBHQ 42%–46%, BHA 44%–47%, BHT 44%–46%	Huang et al. (2018)
PECVD	PBS film	≥ 99% reduction in oxygen transmission rate (OTR)	Vassallo et al. (2022)
PECVD	Stainless steel	1.4 log reduction in viable *Saccharomyces cerevisiae* count after 24 h	Saulou et al. (2009)
PECVD	Flexible PVC film	71.2% blockage of DEHP migration at 100‐nm thickness	Fei et al. (2012)
PECVD	Stainless steel SS316	*Enterobacter sakazakii* adhesion reduced 99.74%; altered Freundlich/Langmuir constants	Şen et al. (2012)
DBD	PLA/EC film	Antibacterial rates of 86% (*E. coli*) and 66% (*S. aureus*)	Hosseini et al. (2022)
DBD	Stainless steel	3.6 to ∼4 log_10_ reduction of *Bacillus subtilis* (Gram positive)	Duday et al. (2013)
DBD	Stainless steel AISI 316	Relative *Listeria monocytogenes* biofilm inhibition 10%–24% (AP10 + AA6, 12°C)	Fernández‐Gómez et al. (2022)
DBD	3D‐printed PLA	Biofilm reduced: 47.7% (*L. monocytogenes*), 50.4% (*P. aeruginosa*), 64.1% (*E. coli*)	Muro‐Fraguas et al. (2020)
DBD	LLDPE film	Surface energy 31.7 → 45.6 mN/m; inhibition of *Latilactobacillus sakei*, *S. aureus*, *Salmonella* Enteritidis	Müller et al. (2022)
DBD	Stainless steel AISI 304	Biofilm reduced up to 44% (*P. aeruginosa*) and 60% (*Candida albicans*); corrosion rate reduced 35%	Getnet et al. (2022)
DBD	PLA film	Water vapor permeability −30%; 6 mm inhibition zone against *S. aureus*	Norozi et al. (2025)
DBD	PE film	100% bacterial reduction against *E. coli* and *S. aureus* (2% chitosan)	Theapsak et al. (2012)
DBD	Kraft paper	Hydrophobic contact angle maintained at 132° ± 8° after continuous immersion in food simulants	Profili et al. (2023)
DBD	Chitosan film	OTR improved to < 0.05 cc/m^2^·day at 60 s	Kongboonkird et al. (2023)
DBD	PE film (DBD pretreatment + SiO*_x_*)	OTR reduced from 700 to ∼70 cc/m^2^·day·atm (> 10‐fold)	H. Li et al. (2019)
DBD	Gelatin/sodium alginate film	Water vapor permeability −42%; TBA 0.54 mg MDA/kg; mesophiles 5.8 log CFU/g	Tahmouzi et al. (2025)

Abbreviations: BHA, butylated hydroxyanisole; BHT, butylated hydroxytoluene; DBD, dielectric barrier discharge; DEDB, dibutyl sebacate; DEHP, di(2‐ethylhexyl) phthalate; DLC, diamond‐like carbon; LLDPE, linear low‐density polyethylene; MDA, malondialdehyde; OTR, oxygen transmission rate; PBS, poly(butylene succinate); PE, polyethylene; PECVD, plasma‐enhanced chemical vapor deposition; PEF, poly(ethylene furanoate); PLA, polylactic acid; PVC, polyvinyl chloride; TBA, thiobarbituric acid; TBHQ, *tert*‐butylhydroquinone; UHMWPE, ultrahigh‐molecular‐weight polyethylene.

**FIGURE 2:**
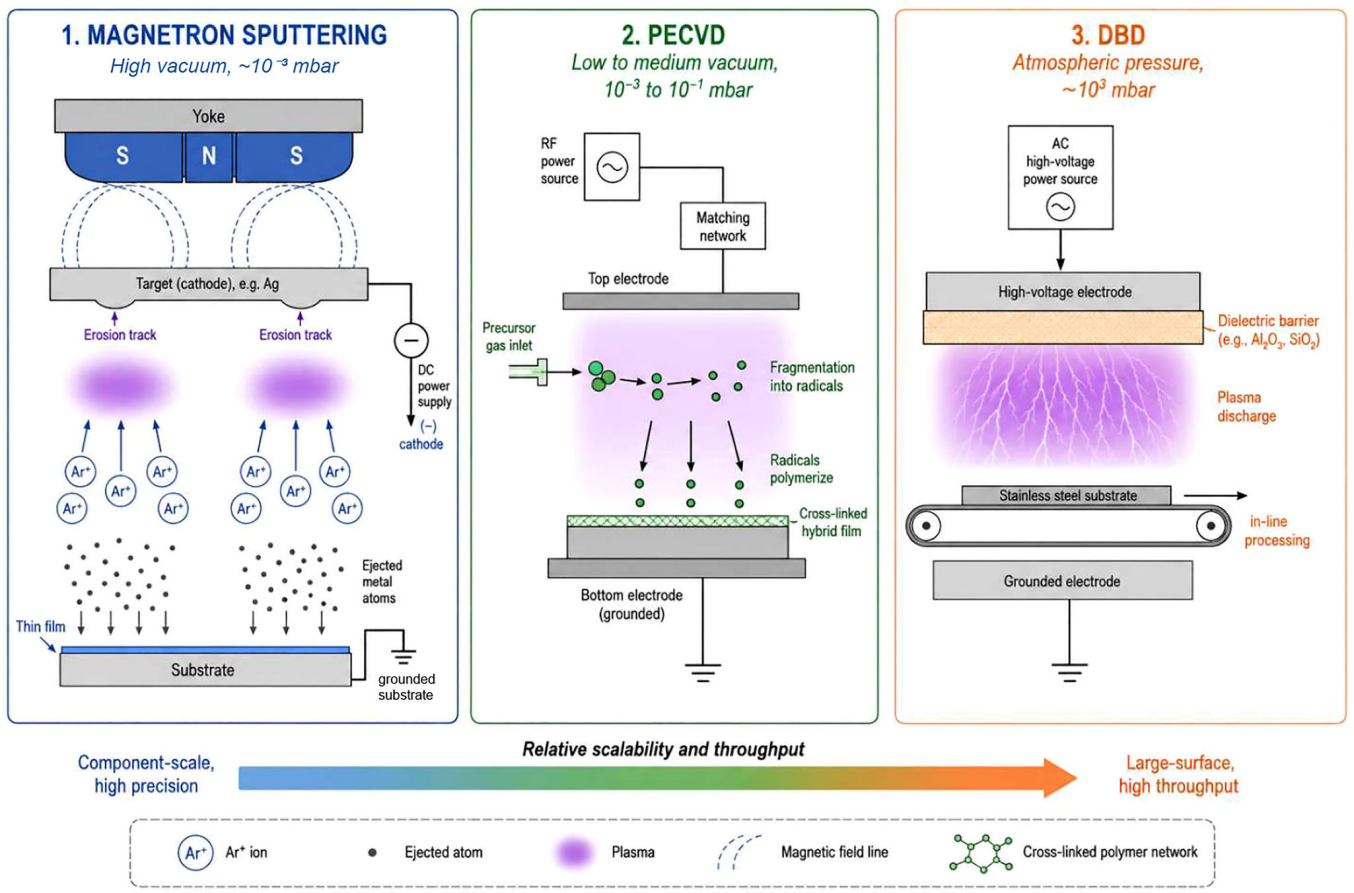
Comparative deposition mechanisms of plasma surface engineering technologies applied to food contact materials. Magnetron sputtering (left) generates ultrathin metallic films through ion bombardment of a target under vacuum, using a center and ring magnet assembly connected by a ferromagnetic yoke to confine secondary electrons in a toroidal field above the target, concentrating plasma density and erosion into a ring and forming controlled‐release ionic reservoirs (e.g., Ag^+^, Zn^2+^). Plasma‐enhanced chemical vapor deposition (PECVD, center) applies radiofrequency power through a matched electrode opposite a grounded electrode, fragmenting and polymerizing gaseous precursors into cross‐linked hybrid films that enable diffusion‐controlled antimicrobial release. Dielectric barrier discharge (DBD, right) operates at atmospheric pressure, with a dielectric layer (e.g., Al_2_O_3_, SiO_2_) positioned between the high‐voltage and grounded electrodes to prevent arc transition, activating surface chemistry and adhesion for subsequently applied coatings without vacuum infrastructure. The three technologies occupy complementary positions along the precision‐to‐scalability continuum. This schematic was generated based on the process mechanisms described in Sections 3.1–3.3 of this review and informed by Bazaka et al. (2015) for magnetron sputtering, Nikiforov et al. (2022) for PECVD, and Özdemir et al. (2023) and Ma et al. (2021) for DBD.

#### 3.4.1 Translational Relevance: Pilot‐Scale and Industrial Implementations

While the majority of plasma surface engineering research remains at the laboratory scale, several pilot‐scale and semi‐industrial implementations provide concrete evidence of translational feasibility under realistic processing conditions. Addressing the challenge of dynamic food contact surfaces, Leipold et al. (2010) integrated an atmospheric pressure DBD system directly onto rotating cutting tools, enabling continuous surface treatment during operation without interrupting the processing line, as later summarized in comprehensive industrial assessments (Katsigiannis et al. 2022). Similarly, Toyokawa et al. (2017) developed a roller conveyor system equipped with a thermally managed nitrogen gas plasma unit for the inline treatment of fresh produce, illustrating the compatibility of plasma‐based surface activation strategies with high‐throughput industrial infrastructure (Katsigiannis et al. 2022; Özdemir et al. 2023).

Beyond food processing equipment, evidence from materials and packaging manufacturing further supports the scalability of plasma‐mediated surface engineering. Štěpánová et al. (2023) validated a roll‐to‐roll diffuse coplanar surface barrier discharge (DCSBD) system capable of modifying polyethylene films at industrial processing speeds of up to 16 cm/s, confirming that plasma‐based interfacial modification can be integrated into continuous manufacturing workflows (Katsigiannis et al. 2022). In parallel, pilot‐scale generation of plasma‐processed air (PPA) and plasma‐activated water (PAW) has been successfully implemented for the bulk treatment of fresh‐cut produce and dried herbs (Durek et al. 2022). These examples support the feasibility of integrating plasma‐based surface engineering into existing industrial workflows, although broader validation under food‐relevant organic loads and established regulatory frameworks remains necessary.

### 3.5 Translational Limits and Integrated Strategies

Despite their complementary strengths, no single plasma technique can fully overcome, in isolation, the thermodynamic and biological constraints imposed by food matrix conditioning films (Flemming et al. 2023). Durable control instead requires integrated interfacial strategies in which hydration‐mediated repulsion, controlled antimicrobial delivery, and mechanical robustness operate synergistically at the bacteria–surface interface (Olatunde et al. 2021).

Beyond thermodynamic constraints, biological complexity further limits the effectiveness of species‐specific antimicrobial surface strategies. In food processing environments, biofilms rarely consist of a single microbial species but rather emerge as structured, multispecies communities embedded within heterogeneous organic matrices (L. Yuan et al. 2020). Within these consortia, interspecies interactions, metabolic cooperation, and matrix‐mediated shielding can attenuate bactericidal mechanisms targeted at individual microorganisms (Flemming et al. 2023; J. Yan and Bassler 2019). As a result, surface technologies designed to act downstream at the microbial level remain vulnerable to ecological variability (Rolon et al. 2024). Plasma‐engineered interfaces designed to modulate matrix–surface interactions and preserve interfacial hydration may operate upstream of microbial diversity, potentially providing a species‐agnostic mechanism to limit early biofilm establishment under realistic industrial conditions, although direct validation in multispecies food processing biofilms remains limited (Hage et al. 2022; Katsigiannis et al. 2022).

Taken together, these thermodynamic and biological constraints indicate that durable microbial control in food processing environments may require integrated, multifunctional surface strategies rather than static or single‐function modifications (Yang et al. 2025). In this context, plasma surface engineering represents a candidate framework for stabilizing and integrating otherwise mechanically fragile surface chemistries, enabling antifouling, antimicrobial, and mechanical functionalities to coexist within a single interfacial architecture. By offering covalent anchoring, graded interfacial architectures, and nanoscale cross‐linking, plasma‐based processes may preserve functional performance under repeated cleaning cycles and continuous organic challenges, supporting the transition from laboratory‐scale demonstrations toward durable industrial implementation (Dufour 2023).

### 3.6 Plasma Surface Engineering in the Context of Alternative Adaptive Surface Strategies

Plasma surface engineering is not the only approach proposed to address food matrix interference on antimicrobial surfaces. Enzymatic antifouling coatings, zwitterionic self‐assembled monolayers (SAMs), biomimetic nanotextured surfaces, and quorum‐sensing inhibition (QSI) coatings each represent conceptually distinct strategies targeting different stages of microbial colonization. A comparative assessment across criteria relevant to industrial food processing, including performance under organic load, durability under CIP cycles, scalability, and regulatory status, is presented in Table 3.

**TABLE 3:** Comparative overview of primary studies on alternative surface strategies against microbial adhesion and biofilm formation on food‐contact materials: enzymatic coatings, quorum‐sensing inhibition (QSI), zwitterionic/PEG‐based surfaces, and biomimetic nanotextured surfaces.

Strategy	Substrate tested	Quantified outcome	References	
Enzymatic	Stainless steel 316L	Lytic activity of 0.5 U against *Micrococcus lysodeikticus* (≈10 ng free‐enzyme equivalent)	Minier et al. (2005)	
Enzymatic	Polystyrene resin beads	Hydrolytic activity 2736 U/g resin (14.2% of free‐enzyme activity)	Wu and Daeschel (2007)	
Enzymatic	Chitosan‐based edible coating	72.5% enzyme activity retained (145,000 U/g chitosan); inhibition zones against *Escherichia coli* O157:H7 and *Staphylococcus aureus*	Lian et al. (2012)	
Enzymatic	LDPE/HDPE/LLDPE/PCL packaging films	9‐log reduction of *Acinetobacter* sp. and *S. aureus* on beef after 7 days at 4°C	Manohar et al. (2015)	
Enzymatic	LDPE coupons	70.7 ± 5.0% reduction in adhered *E. coli*; 81.8 ± 16.7% reduction in biofilm thickness	Cattò et al. (2018)	
Enzymatic	Stainless steel AISI 316	54% reduction in biofilm‐occupied area at PRN/BAC 1000/2000 µg/mL; *E. coli* below detection limit, possible VBNC persistence	Rodríguez‐López et al. (2017)	
QSI	Polyurethane coating	Anti‐biofilm activity via immobilized acylase (AHL degradation)	Grover et al. (2016)	
QSI	PVDF/graphene oxide membrane	Enhanced anti‐biofouling via acylase‐based quorum quenching	Z. Zhu et al. (2018)	
QSI	Cu‐bearing stainless steel 304 L	54.78% reduction in *E. coli* biofilm biomass; 34.35% reduction in EPS coverage	X. Zhang, Sun, et al. (2024)	
QSI	Stainless steel	1.5–3 log CFU/cm^2^ reduction in *Pseudomonas aeruginosa* biofilm; pyocyanin reduced up to 60%	Tapia‐Rodriguez et al. (2017)	
QSI	Stainless steel coupons and hand gloves	0.10–2.17 log CFU/cm^2^ reduction (SS); suppressed *aphA*/*luxS* QS gene expression	Roy et al. (2022)	
Zwitterionic/PEG	Stainless steel AISI 316	Best case within 76%–90% reduction range at 12°C (AP10 + AA6), same dataset as Table 4, ^a^	Fernández‐Gómez et al. (2022)	
Zwitterionic/PEG	Stainless steel AISI 316	*Enterobacter sakazakii* adhesion reduced 99.74% (EDA)/to 375 CFU/cm^2^ (PEGMA)	Şen et al. (2012)	
Zwitterionic/PEG	UHMWPE	4.8 log (*E. coli*) and 2.1 log (*S. aureus*) reduction	Zabihzadeh Khajavi et al. (2024)	
Zwitterionic/PEG	Au/SiO_2_ model surface (OEG SAM)	Prevented β‐lactoglobulin fouling via hydration repulsion	Skoda et al. (2022)	
Zwitterionic/PEG	Stainless steel	PEG‐like plasma coating significantly reduced mixed‐culture biofilm formation vs. unmodified steel	Denes et al. (2001)	
Zwitterionic/PEG	Stainless steel	(vs. PET–PEG comparator) SS‐PEG failed to reduce *Pseudomonas* sp. adhesion vs. control; PET–PEG reduced adhesion 2–4 orders of magnitude	Kingshott et al. (2003)	
Zwitterionic/PEG	Stainless steel	SI‐ATRP PEGMA brushes with lysozyme coupling prevented BSA adsorption and reduced *E. coli*/*S. aureus* adhesion and biofilm formation	S. Yuan et al. (2011)	
Zwitterionic/PEG	Polyamide/polyester	(PA‐PEG, PET–PEG) Plasma‐grafted PEG confirmed by ESCA; reduced bacterial adhesion (*S. aureus*, *P. aeruginosa*) vs. unmodified substrate	Dong et al. (2007)	
Zwitterionic/PEG	Stainless steel	Silane‐coupled PEG grafting significantly reduced protein adsorption vs. unmodified steel	F. Zhang et al. (2001)	
Biomimetic	Stainless steel STS316L	> 99.9% inhibition (*E. coli*, *S. aureus*, *P. funiculosum*); safe Zn elution, 27.2 µg/L	Shim et al. (2017)	
Biomimetic	3D‐printed PLA	Biofilm reduced 47.7%–64.1% (*L. monocytogenes*, *P. aeruginosa*, *E. coli*)	Muro‐Fraguas et al. (2020)	
Biomimetic	PMMA nanopillar arrays	Reduced *E. coli* adhesion (P1100 pitch); bactericidal effect (P480 pitch)	Heckmann and Schiffman (2020)	

Abbreviations: AHL, acyl‐homoserine lactone; BAC, benzalkonium chloride; EPS, extracellular polymeric substances; HDPE, high‐density polyethylene; LDPE, low‐density polyethylene; LLDPE, linear low‐density polyethylene; OEG, oligo(ethylene glycol); PCL, polycaprolactone; PEG, polyethylene glycol; PLA, polylactic acid; PMMA, poly(methyl methacrylate); PVDF, polyvinylidene fluoride; QSI, quorum‐sensing inhibition; SAM, self‐assembled monolayer; UHMWPE, ultrahigh‐molecular‐weight polyethylene.

^a^ Fernández‐Gómez et al. (2022) derived values from the same experimental dataset (AP10 + AA6 coating, 12°C, 3 strains, 144–288 h); 90% represents the best‐case reduction within the 76%–90% range, not an independent condition. At 37°C, the coating showed a pro‐biofilm effect in some of the strains (relative production 34%–177%).

Across the strategies evaluated, a consistent pattern emerges: technologies that perform well under controlled laboratory conditions tend to lose functionality rapidly when exposed to the organic complexity and mechanical demands of industrial food processing environments. Enzymatic and QSI coatings are constrained by molecular fragility and leaching, zwitterionic SAMs by interfacial masking, and biomimetic surfaces by topographic fouling. Plasma surface engineering addresses several of these limitations through covalent interfacial anchoring, dry deposition compatible with complex geometries, and the capacity to integrate antifouling and antimicrobial functionalities within a single film architecture. However, it shares with all competing strategies the unresolved challenge of long‐term validation under realistic CIP cycles and food‐relevant organic loads and faces additional regulatory requirements specific to metal‐releasing and nanostructured food contact materials (Katsigiannis et al. 2022; Chowdhury et al. 2025).

### 3.7 Limitations, Durability, and Regulatory Constraints

The translational potential of plasma surface engineering must be assessed alongside its inherent limitations, which bear direct industrial applicability. One of the primary challenges concerns long‐term durability under humid, chemically aggressive food processing environments (Katsigiannis et al. 2022). Plasma‐polymerized coatings containing organic functionalities may undergo hydrolytic degradation under prolonged exposure to water, cleaning agents, and temperature cycling when the cross‐linking density is insufficient (Bharathi et al. 2025). Inadequate process control can also result in excessive etching or surface embrittlement, particularly when treating polymeric substrates (Akdoğan and Şirin 2021).

Another well‐documented limitation is surface aging, commonly referred to as hydrophobic recovery (the gradual loss of surface wettability due to molecular reorientation or adsorption of low‐energy species). Without appropriate chemical stabilization or post‐deposition cross‐linking, this phenomenon compromises long‐term antifouling performance (S. Sharma et al. 2022).

From an industrial perspective, cost and scalability remain relevant constraints. Vacuum‐based plasma technologies, such as magnetron sputtering and PECVD, require capital‐intensive infrastructure and are less suited for the treatment of very large components or enclosed systems (Sanyal Dipto et al. 2023). While atmospheric plasma approaches offer improved scalability, they provide limited control over nanoscale architecture and typically function as complementary pretreatment steps rather than standalone solutions (R. Sharma et al. 2025).

The dimensional constraints of vacuum chambers do not preclude the practical use of sputtering and PECVD but rather define their optimal domain of application. These technologies are inherently impractical for the in situ treatment of large, fixed industrial infrastructure (Katsigiannis et al. 2022; Sanyal Dipto et al. 2023). However, their industrial viability relies on a stratified application strategy targeting critical modular components with an elevated risk of biofilm persistence. High‐risk loci, including heat exchanger plates, valve assemblies, and industrial cutting tools, can be treated ex situ with durable nanostructured coatings before installation or during scheduled maintenance intervals (Leipold et al. 2010). In parallel, atmospheric‐pressure plasma (APP) technologies, such as DBD, provide the scalability required for in situ surface activation and interfacial preparation of large conveyor systems and fixed processing lines (Özdemir et al. 2023). Within this hybrid framework, vacuum and atmospheric plasma technologies operate as complementary elements of an integrated surface engineering strategy, ensuring functional coverage across both component‐level and infrastructure‐scale applications.

Regulatory approval represents an additional and often underestimated barrier to industrial deployment. In the European Union, plasma‐engineered food contact materials must comply with Regulation (EC) No 1935/2004, which establishes inertness requirements and prohibits the transfer of constituents to food at levels that may endanger human health. Metal‐releasing coatings, such as those incorporating Zn^2+^ or Ag^+^, are subject to specific migration limits evaluated by the European Food Safety Authority (EFSA), which has issued dedicated guidance on the safety assessment of nanomaterials in food contact applications. In the United States, plasma‐deposited surface modifications intended for food contact fall under FDA jurisdiction through the Food Contact Substance Notification (FCN) process under 21 CFR, requiring characterization of migrating species, estimated dietary exposure, and toxicological risk assessment. A specific concern for plasma‐engineered coatings is cleaning compatibility: repeated exposure to alkaline CIP agents, chlorine‐based sanitizers, and acidic rinses may alter surface chemistry, accelerate ion leaching beyond regulatory thresholds, or generate degradation products not covered by existing approvals. Systematic migration testing under food‐simulating conditions combined with accelerated CIP cycling protocols will be necessary to generate the safety dossiers required for regulatory clearance in both jurisdictions (Alaguthevar et al. 2024; Sanyal Dipto et al. 2023).

These limitations collectively define the conditions under which plasma surface engineering can be rationally deployed: successful implementation requires careful process optimization, integration of complementary plasma techniques, and systematic performance evaluation under industrial CIP conditions (Hadinoto et al. 2023).

### 3.8 Techno‐Economic and Sustainability Considerations

The industrial adoption of plasma‐engineered antimicrobial surfaces is frequently perceived as constrained by high initial capital expenditure (CAPEX). However, when evaluated through a techno‐economic framework that integrates operational expenditures (OPEX), energy efficiency and risk mitigation, plasma‐based surface engineering can be economically competitive and, in several scenarios, favorable relative to conventional sanitation (R. Sharma et al. 2025). Although absolute cost comparisons vary depending on facility size, process configuration and plasma modality, order‐of‐magnitude estimates provide a realistic basis for assessing industrial viability (Katsigiannis et al. 2022).

#### 3.8.1 Operational Cost Efficiency vs. Chemical Sanitation

Conventional sanitation strategies based on alkaline and acidic agents carry low upfront costs, largely due to their widespread availability and established integration into existing CIP protocols. However, these approaches impose recurring operational expenses associated with continuous chemical consumption, water and energy use, equipment corrosion, production downtime, and waste management (Chowdhury et al. 2025). When evaluated over the full lifecycle of food contact surfaces, such recurring costs challenge assessments based solely on initial investment (Alaguthevar et al. 2024).

While the upfront investment associated with plasma systems may appear substantial, life‐cycle assessments consistently indicate a reduction in long‐term operational costs relative to conventional chemical sanitation. Industrial‐scale estimates for atmospheric plasma systems report electricity costs on the order of $4500 per 1000 h of operation, a value that is lower than the recurring expenses associated with high‐grade chemical disinfectants, hazardous material handling and wastewater treatment requirements (Katsigiannis et al. 2022; Mostashari et al. 2022).

In the food processing sector, annual expenditures related to compliance with traditional sanitation protocols are estimated to range between $520 and $860 million in the United States alone, with additional operational losses attributed to extended CIP procedures and production downtime. Aggressive chemical sanitation cycles are also associated with opportunity losses exceeding $10 million per year in large processing facilities (Chowdhury et al. 2025; Katsigiannis et al. 2022).

When plasma technologies are applied to engineer durable, matrix‐repellent or easy‐to‐clean surfaces, the primary economic benefit does not arise from continuous plasma operation, but from reduced chemical consumption, shorter sanitation cycles and decreased downtime (Hua and Zhu 2024a). In this context, plasma surface engineering may redirect sanitation economics from repetitive chemical intervention toward preventive interfacial control, contingent on demonstrated long‐term durability under industrial conditions (Dufour 2023).

#### 3.8.2 Energy Efficiency and Low‐Carbon Processing

Plasma‐based technologies also compare favorably in energy efficiency. Cold plasma pretreatment has been shown to reduce energy consumption in food processing, with reported energy savings ranging from approximately 20% (e.g., reducing consumption from 53.4 to 42.6 kWh/kg in specific drying processes) to 40% in targeted decontamination protocols (R. Sharma et al. 2025). In optimized hybrid systems combining plasma with hot air, reductions in processing time from 9600 to 3600 s have been reported, corresponding to an approximately 2.7‐fold reduction in processing duration relative to traditional thermal drying (Gavahian et al. 2024; R. Sharma et al. 2025).

In addition to energy savings, plasma‐assisted sanitation offers substantial reductions in water usage. The replacement of chemical‐based protocols with ozone‐ or plasma‐derived treatments in beverage processing systems, including wineries, has been shown to reduce water consumption by approximately 98 million liters per year in large‐scale operations (Mostashari et al. 2022). These reductions simultaneously decrease the energy demand for water heating and minimize chemical effluent generation, aligning plasma‐engineered surfaces with low‐carbon and sustainability‐driven food production strategies (Gavahian et al. 2024; Hadinoto et al. 2023).

#### 3.8.3 Macro‐Economic Impact of Biofilm Mitigation

The costs associated with biofilm persistence in industrial environments provide an additional economic context for plasma‐engineered surfaces. The global economic burden of biofilms in the agri‐food sector alone is estimated at approximately $324 billion annually, within a broader estimated economic impact of biofilms exceeding $5 trillion across all industrial sectors combined (Cámara et al. 2022). In food processing environments, persistent biofilms contribute not only to equipment degradation, but also to product recalls, production shutdowns and long‐term brand damage, with individual recall events frequently costing millions of dollars (Alonso et al. 2023).

By potentially limiting initial microbial attachment and modulating organic conditioning film formation at the interface, plasma‐engineered surfaces may function as a preventive complement to established reactive sanitation measures (Hage et al. 2022). In addition to reducing contamination risk, such surfaces have been associated with extended product shelf life, reduced food waste and improved process reliability (Alaguthevar et al. 2024; Y. Zhao et al. 2023). From a macroeconomic perspective, the potential of plasma surface engineering to reduce recall frequency, sanitation downtime, and resource consumption supports its evaluation as a preventive complement to established sanitation programs, provided that performance is validated under realistic industrial conditions.

### 3.9 Critical Synthesis of Section 3

Taken together, while plasma surface engineering offers clear techno‐economic advantages and multiscale deposition control, its industrial adoption remains constrained by a persistent engineering trade‐off: vacuum‐based technologies (PECVD, sputtering) deliver superior nanostructural integrity but require high capital investment for modular components (Sarakinos et al. 2010), whereas atmospheric systems (DBD) provide operational scalability at the expense of precise nanostructural tuning (as detailed in Table 2), particularly for large or geometrically complex equipment where vacuum chamber configurations become technically challenging and economically prohibitive (S. Liu et al. 2022). The primary challenge moves beyond proving immediate biocidal efficacy to establishing long‐term coating survival under aggressive CIP regimens (van den Berg et al. 2024) and navigating complex EFSA/FDA regulatory frameworks regarding nanomaterial leaching (Vaidulych et al. 2017; Chowdhury et al. 2025; Katsigiannis et al. 2022). Bridging this translational gap requires a shift from single‐function surface treatments toward hybrid, multitiered architectures that couple covalent plasma anchoring with dynamic, diffusion‐controlled ion release (Xu et al. 2019; Schlebrowski et al. 2021). Understanding how these plasma‐modified interfaces alter downstream bacterial sensing and molecular stress responses (Linklater et al. 2021) is a prerequisite for designing durable, matrix‐resilient food contact materials.

## 4 Strategic Integration of Surface Chemistry and Matrix Interference

### 4.1 Hydration‐Mediated Repulsion and Antifouling Mechanisms

Among antifouling strategies, hydration‐mediated repulsion is consistently reported as one of the most effective mechanisms for suppressing nonspecific protein adsorption. Zwitterionic and highly hydrophilic surfaces exemplify this principle by stabilizing dense interfacial water layers through strong ionic solvation and hydrogen bonding (B. Song et al. 2024). In these systems, tightly bound hydration layers create a substantial energetic barrier to macromolecular adsorption (Choi et al. 2020).

From a thermodynamic perspective, hydration barriers directly counter the entropy‐driven adsorption mechanisms described in Section 2. For macromolecules to adsorb, dehydration of the interface must first occur, imposing a substantial energetic penalty (Q. Li et al. 2022). As a result, zwitterionic surfaces frequently exhibit near‐zero protein adsorption under controlled laboratory conditions and are widely regarded as the physicochemical benchmark for antifouling performance (Mu et al. 2023).

Hydration‐based surfaces have not achieved widespread industrial adoption, however, primarily due to the susceptibility of zwitterionic monolayers and hydrogels to mechanical abrasion, chemical degradation, and limited interfacial adhesion to stainless steel substrates (S. Liu et al. 2022). Under repeated CIP cycles, these coatings often lose structural integrity or hydration capacity. Zwitterionic surfaces define an ideal antifouling state but fail as standalone solutions in food processing environments (Chowdhury et al. 2025).

### 4.2 Industrial Limitations of Hydration‐Based Surfaces

The dominant antifouling literature often assumes static interfaces operating in controlled aqueous environments. In contrast, industrial food contact surfaces experience dynamic conditions, including fluctuating pH, temperature cycling, mechanical shear, and continuous exposure to heterogeneous organic loads (Katsigiannis et al. 2022). Under such conditions, even advanced hydration‐based coatings gradually lose effectiveness (Chowdhury et al. 2025).

Moreover, hydration‐mediated repulsion alone does not prevent the long‐term accumulation of organic debris. Partial masking by lipids or denatured proteins can locally disrupt hydration layers, creating defects that act as nucleation sites for bacterial attachment (Alonso et al. 2023; Q. Li et al. 2022). Once colonization initiates at these sites, the antifouling advantage rapidly diminishes (Alonso et al. 2023).

These observations indicate that antifouling and antimicrobial strategies cannot be treated as independent or mutually exclusive. Hydration barriers delay fouling but do not eliminate it indefinitely (J. H. Cheng et al. 2025). Effective surface designs must therefore integrate passive repulsion with active defense mechanisms capable of functioning beneath partially formed conditioning films (S. Sharma et al. 2022).

### 4.3 Plasma Surface Engineering as a Translational Enabler

Plasma surface engineering provides a pathway to translate hydration‐mediated antifouling concepts from laboratory demonstrations into industrially viable systems (Cazalini et al. 2017). Classical zwitterionic monolayers remain the benchmark under idealized conditions, but plasma technologies do not seek to replicate these chemistries identically (S. Liu et al. 2022). Instead, they translate the underlying hydration functionality into mechanically robust and scalable interfacial architectures (Ma et al. 2021).

Through PECVD, hydrophilic or zwitterionic functionalities can be covalently incorporated into highly cross‐linked plasma‐polymerized networks (Bertran‐Serra et al. 2023). This architecture significantly enhances mechanical stability and adhesion to stainless steel substrates, overcoming a primary limitation of conventional antifouling coatings (Múgica‐Vidal et al. 2019). By anchoring hydration‐promoting chemistries directly to the substrate, plasma processing preserves hydration capacity under mechanical and chemical stress (Bertran‐Serra et al. 2023).

Plasma techniques also enable the fabrication of multifunctional coatings. Hydration‐mediated repulsion can be combined with embedded antimicrobial reservoirs, yielding interfaces that resist initial protein adsorption while retaining antimicrobial functionality once fouling becomes unavoidable (S. Liu et al. 2020). This dual‐mode behavior extends the functional range beyond that of purely static antifouling approaches.

### 4.4 Controlled Release Systems Beneath the Conditioning Film

Recognizing that no surface remains pristine indefinitely in industrial environments, plasma surface engineering enables the design of controlled release systems that operate beneath adsorbed organic layers. Antimicrobial ions such as Zn^2+^ and Ag^+^ can be immobilized within plasma‐polymerized matrices, allowing diffusion‐controlled delivery rather than uncontrolled leaching (Deng et al. 2015).

Plasma deposition induces the formation of a dense, cross‐linked superficial layer commonly referred to as the CASING effect (cross‐linking by activated species of inert gases) (Hagiwara et al. 2013). By preventing rapid depletion and excessive ion flux, this architecture enhances long‐term efficacy while mitigating toxicological concerns (Deng et al. 2015).

Furthermore, plasma‐engineered systems can be designed to respond to environmental cues associated with microbial activity. Experimental evidence indicates that ion release rates may increase under acidic conditions, reflecting the localized acidification characteristic of early biofilm metabolism (Buchegger et al. 2019).

This mechanism is directly applicable in food processing environments where organic conditioning films are unavoidable. While contact‐dependent antimicrobial coatings may be rapidly masked by proteins and lipids, diffusible ions may retain the capacity to penetrate these layers and preserve antimicrobial activity beneath the organic interface (R. Sharma et al. 2025).

### 4.5 Adaptive Interfaces Under Thermodynamic Constraints

The integration of hydration barriers, controlled antimicrobial release, and mechanically robust surface anchoring transforms food contact materials from passive substrates into adaptive interfacial systems. Rather than attempting to oppose entropy‐driven phenomena, plasma‐engineered interfaces are explicitly designed to operate within them (Q. Li et al. 2022).

This framework reconciles the longstanding discrepancy between laboratory‐scale performance and industrial‐scale failure (S. Sharma et al. 2022). While zwitterionic chemistries represent the physicochemical benchmark for antifouling behavior under idealized and clean conditions, plasma‐based processing provides the structural durability, adhesion strength, and functional persistence required for deployment in heterogeneous, aged food matrices (Akdoğan and Şirin 2021). Together, these approaches reduce the gap between reductionist experimental models and the complexity of real food processing environments (Chowdhury et al. 2025).

By embedding antifouling and antimicrobial functionalities directly into the material interface, plasma‐engineered surfaces reduce dependence on intensive chemical sanitation, mitigate surface degradation over repeated cleaning cycles, and align with increasingly stringent regulatory and environmental constraints (Costa et al. 2023). This strategy frames microbial control as an interfacial design problem grounded in thermodynamics, surface physics, and microbial ecology, complementing rather than replacing reactive sanitation measures (Bharathi et al. 2025).

### 4.6 Conceptual Synthesis and Design Implications

The central conclusion of this section is that neither plasma treatment nor zwitterionic chemistry alone constitutes a complete solution. Plasma applied solely as a surface activation tool provides transient benefits (Mozetič 2023), while zwitterionic coatings without structural reinforcement fail under industrial stress. Effective antimicrobial surfaces emerge from their deliberate integration.

By stabilizing hydration barriers, enabling controlled antimicrobial delivery, and ensuring mechanical durability, plasma surface engineering transforms theoretical antifouling concepts into operational technologies (Sharifahmadian et al. 2021). This integrated perspective treats antimicrobial surface design as a systems problem in which thermodynamics, surface physics, and microbiology converge (Kimkes and Heinemann 2019).

Section 5 examines how bacteria sense and respond to these engineered interfaces at the molecular and regulatory levels (Qin and Bassler 2022).

Collectively, these considerations demonstrate that antimicrobial strategies differ fundamentally in how they interact with food matrix dynamics. While conventional sanitizers, contact‐active coatings and laboratory‐scale antifouling materials each address isolated aspects of microbial control, they remain vulnerable to organic masking, sublethal stress gradients and limited mechanical durability (Katsigiannis et al. 2022). Plasma‐engineered adaptive surfaces aim to integrate hydration‐mediated repulsion, mechanotransduction modulation, and controlled antimicrobial delivery within a single interfacial system, although the simultaneous optimization of these functions under industrial conditions remains an active area of investigation. A comparative synthesis of these approaches is presented in Table 4, which compiles primary studies quantifying residual persistence and VBNC induction across chemical sanitizers, contact‐killing coatings, antifouling chemistries, and plasma‐based strategies on food contact substrates.

**TABLE 4:** Comparative overview of primary studies evaluating persistence and viable but non‐culturable (VBNC) induction across antimicrobial surface strategies in food‐contact environments.

Strategy	Substrate/pathogen	Quantified outcome	References
Chemical sanitizer	Chicken skin, food‐contact surfaces/*Salmonella* Typhimurium	PAA showed higher efficacy than chlorine‐based disinfectants	Mahamud et al. (2025)
Chemical sanitizer	Dairy pipeline stainless steel/*Listeria monocytogenes*, *Flavobacterium* spp.	CIP reduced counts 1–2 logs; additional sanitization step not significantly effective (*p* > 0.05)	Bremer et al. (2006)
Chemical sanitizer	Stainless steel and PVC/*L. monocytogenes* + microbiota	≤ 4.5 log reduction (SS, single‐species); 1.2–2.4 log reduction (PVC, multispecies, with regrowth)	Lake et al. (2024)
Chemical sanitizer	Worn SS‐2B and LDPE (apple juice residue)/*L. monocytogenes*	QAC: 1.38 log (worn) vs. 2.21 log (clean); PAA: 2.58–2.78 log (worn) vs. 3.24–3.50 log (clean)	Hua and Zhu (2024b)
Chemical sanitizer	Stainless steel 304 and PP‐B/*L. monocytogenes*, *P. aeruginosa*, *Escherichia coli*, *Salmonella*	QAC: 6 log reduction after 24 h; efficacy modulated by organic matter type (meat extract vs. egg yolk/milk)	Iñiguez‐Moreno et al. (2018)
Chemical sanitizer	Stainless steel/*Vibrio parahaemolyticus*	Residual culturable population 1.25 log CFU/cm^2^; VBNC fraction increased up to 7.20 Δlog	Régnier et al. (2025)
Chemical sanitizer	Polyurethane conveyor coupons/*E. coli* O157:H7	VBNC induction successful at high initial biofilm population (6.7 log CFU/cm^2^), not at low (5.4 log CFU/cm^2^)	Marouani‐Gadri et al. (2010)
Chemical sanitizer	Stainless steel and PVC/*L. monocytogenes*	5.22 log (H_2_O_2_) and 4.49 log (QAC) culturability reduction; VBNC detected at 2.95 and 2.12 log GE/cm^2^	Brauge et al. (2020)
Chemical sanitizer	Stainless steel and ceramic/*L. monocytogenes*	Sublethal daily sanitizer exposure induced sessile VBNC population	Overney et al. (2017)
Chemical sanitizer	Stainless steel and PP/*Salmonella* spp.	Culturable cells < 0.7 log CFU/cm^2^ post‐ethanol; VBNC cells remained above detection limit	Prestes et al. (2025)
Chemical sanitizer	Stainless steel AISI 316 (dual‐species biofilm)	At low BAC concentration, pronase co‐treatment showed pro‐biofilm effect: occupied area 0.64 vs. 0.51 mm^2^ without pronase; *E. coli* release increased 4.90–5.29 log CFU/mL	Rodríguez‐López et al. (2017)
Chemical sanitizer	Buna‐N, Viton, EPDM, silicone, Teflon (aged vs. new)/*L. monocytogenes*	Aged elastomers: 0.6–1.4 log reduction (QAC/chlorine); PAA: 3.1 log (Buna‐N), 2.7 log (EPDM)	Thapa et al. (2026)
Chemical sanitizer	Stainless steel/*E. coli* O157:H7	HPS‐induced VBNC; resuscitation achieved within 24 h	K. Zhao et al. (2026)
Chemical sanitizer	Stainless steel (flow cell, turbulent/laminar flow)/*Pseudomonas fluorescens*	Biofilm mass increased +33% (2 h) and +20% (12 h) post‐CTAB, indicating VBNC‐related regrowth	Araújo et al. (2017)
Chemical sanitizer	Stainless steel (meat broth soiling)/*E. coli* O157:H7	Combined gallic acid + disinfectant increased inhibition 94.11% (QAC) and 91.49% (ClO_2_) vs. sanitizer alone	C. Zhang, Li, et al. (2024)
Contact‐killing coating	Metal sheets (silane QAC spray)/*Listeria innocua*, *E. coli* K12	> 5 log reduction sustained through 20 wash cycles (*L. innocua*); ∼2 log through 5 cycles (*E. coli*)	Lee and Pascall (2020)
Contact‐killing coating	LDPE (N‐halamine)/*E. coli* O157:H7, *L. innocua*	5 log reduction; withstood 5 rechlorination cycles (20 ppm); full removal after 50°C wash	Zou et al. (2024)
Contact‐killing coating	Stainless steel and polyurethane/*Salmonella*, *L. monocytogenes*	Clean: 4.6 log (*Lm*), 3.7 log (*Se*); high organic load: reduced to 0.7 log (*Lm*), 1.2 log (*Se*)	Kalb et al. (2023)
Contact‐killing coating	Stainless steel (nisin‐immobilized)/*Bacillus subtilis*	3.6 log reduction, stable after 10 abrasive cleaning cycles/24 h detergent immersion	Duday et al. (2013)
Contact‐killing coating	Polyethylene (chitosan‐coated)/*E. coli*, *Staphylococcus aureus*	100% bacterial reduction maintained after three forced washing cycles	Theapsak et al. (2012)
Contact‐killing coating	Gelatin/sodium alginate film (AgNPs)/*E. coli* O157:H7, *Salmonella* Typhimurium	Mesophile counts maintained at 5.8 log CFU/g on Day 9 (trout fillet storage)	Tahmouzi et al. (2025)
Contact‐killing coating	LLDPE/cassava starch (ZnO‐NPs)/*S. aureus*, *Salmonella* Enteritidis, *Latilactobacillus sakei*	6.37 log (*S. aureus*) and 6.46 log (*S*. Enteritidis) reduction in complex organic matrix	Müller et al. (2022)
Contact‐killing coating	Stainless steel 316L (Ag/organosilicon nanocomposite)/*Saccharomyces cerevisiae*	τW50%: 1.8 Pa coated vs. no detachment at 100 Pa bare steel; retained after cleaning despite 82% Ag loss. No biocidal effect (viability unchanged, 24 h)	Guillemot et al. (2008)
Zwitterionic/PEG	UHMWPE/*E. coli*, *S. aureus*	4.8 log (*E. coli*) and 2.1 log (*S. aureus*) reduction under diluted nutrient broth (ISO 22196)	Zabihzadeh Khajavi et al. (2024)
Zwitterionic/PEG	Stainless steel AISI 316/*L. monocytogenes* (persistent strains), *E. coli*	AP10 + AA6, 12°C: 10%–24% relative biofilm production (3 strains, 144–288 h) ≡ 76–90% reduction vs. uncoated steel; best case 90%^a^	Fernández‐Gómez et al. (2022)
Zwitterionic/PEG	Stainless steel AISI 316/*E. sakazakii*	PEGMA: 375 vs. 9666 CFU/cm^2^ (bare steel); EDA: 99.74% reduction	Şen et al. (2012)
Plasma‐engineered surface	Stainless steel (PECVD SiO*_x_*/DLC, TiC ion implantation)/*Bacillus cereus*, *B. subtilis* spores	Residual spores 2.15 × 10^1^ – 5.43 × 10^3^ CFU/cm^2^ after 12 fouling/CIP cycles	Rosmaninho, Rocha, et al. (2007)
Plasma‐engineered surface	Stainless steel (APP nitrogen plasma)/*L. monocytogenes*	Culturable cells largely inactivated; VBNC induction confirmed via 16S qPCR and resuscitation assay	Alessandria et al. (2019)

Abbreviations: AgNPs, silver nanoparticles; CIP, cleaning‐in‐place; CTAB, cetyltrimethylammonium bromide; DLC, diamond‐like carbon; GE, genome equivalents; HPS, hydrogen peroxide‐silver; LDPE, low‐density polyethylene; LLDPE, linear low‐density polyethylene; PAA, peracetic acid; PECVD, plasma‐enhanced chemical vapor deposition; PP, polypropylene; QAC, quaternary ammonium compound; TiC, titanium carbide; UHMWPE, ultrahigh‐molecular‐weight polyethylene; VBNC, viable but non‐culturable; ZnO‐NPs, zinc oxide nanoparticles.

^a^ Fernández‐Gómez et al. (2022) derived values from the same experimental dataset (AP10 + AA6 coating, 12°C, 3 strains, 144–288 h); 90% represents the best‐case reduction within the 76%–90% range, not an independent condition. At 37°C, the coating showed a pro‐biofilm effect in some of the strains (relative production 34%–177%).

Despite the conceptual elegance of integrating hydration barriers with diffusion‐controlled ion release, a critical operational bottleneck lies in precise release kinetics management beneath organic conditioning layers. As highlighted in Table 4, improperly calibrated ion elution profiles run the severe risk of establishing sublethal concentration gradients within dense food matrix deposits, inadvertently triggering envelope stress responses or inducing VBNC states in persistent pathogens such as *Salmonella*. To overcome this limitation, translational efforts must move beyond static coating formulations toward responsive interfacial architectures capable of modulating biocidal flux in direct response to localized microenvironmental cues, such as pH drops or enzymatic cleavage by microbial matrix components. Elucidating how bacterial mechanosensors and global transcriptional regulators process these interfacial physical signals is a prerequisite for designing stress‐resilient food contact materials that prevent dormancy and ensure complete microbial eradication under industrial conditions.

## 5 Molecular Responses to Engineered Interfaces: Mechanotransduction and Regulatory Suppression

While Sections 2, 3, 4 established the interfacial, physicochemical, and technological framework governing antimicrobial surface performance, the ultimate success or failure of these strategies is dictated by bacterial perception and response at the molecular and regulatory levels (Bharathi et al. 2025). Bacterial adhesion and biofilm formation are not passive physicochemical events but are tightly regulated biological processes driven by surface sensing, signal transduction and coordinated gene expression (Waters and Bassler 2005). Classical surface science models often treat bacteria as passive colloidal entities governed by classical DLVO‐type interactions (Derjaguin, Landau, Verwey, and Overbeek), which explain adhesion based solely on the physical balance between attractive van der Waals forces and electrostatic repulsion. However, by treating the bacterium as an inert particle, such models neglect the active mechanotransduction and biological appendages that allow pathogens to sense and bypass these physical barriers (Mu et al. 2023).

Although mechanistic insights are frequently derived from model organisms such as *Pseudomonas aeruginosa* and *Escherichia coli*, the regulatory pathways discussed in this section, including surface mechanotransduction, cyclic di‐GMP (c‐di‐GMP) signaling and the RpoS–CsgD regulatory axis, are highly conserved in *Salmonella* spp. and are directly relevant to persistence in food processing environments (Islam et al. 2024). Understanding how engineered surfaces may interfere with these conserved pathways is therefore relevant to evaluating whether plasma‐based strategies can suppress long‐term persistence rather than only transiently delaying surface colonization (Z. Xu et al. 2024).

### 5.1 Surface Sensing and Mechanotransduction

Initial bacterial attachment is mediated by active environmental sensing through cellular appendages, including flagella and Type IV pili, which function as mechanotransducers converting physical surface cues, such as topography and apparent stiffness, into intracellular biochemical signals (Doig et al. 1988; Zheng et al. 2021). Upon surface contact, the flagellum interacts with the substrate, generating viscous drag and mechanical load on the MotA/MotB stator complex. Restriction of flagellar rotation elevates intracellular concentrations of the second messenger c‐di‐GMP, acting as a mechanical trigger for biofilm initiation (F. Song et al. 2018).

Increased c‐di‐GMP concentration functions as a molecular switch, repressing motility while activating transcriptional programs associated with irreversible attachment and biofilm maturation (Condinho et al. 2023). This transition from a planktonic to sessile lifestyle precedes extracellular polymeric substance production and marks commitment to surface colonization (Rabin et al. 2015).

In *Salmonella* spp., this mechanotransductive cascade has been directly documented at the molecular level. Upon surface contact, *S*. Typhimurium suppresses Class III flagellar gene expression and upregulates diguanylate cyclase activity through AdrA, a GGDEF‐domain enzyme whose transcription is directly controlled by CsgD (Shi and Zhu 2009; Steenackers et al. 2012). Elevated c‐di‐GMP concentrations downstream of AdrA activation promote cellulose biosynthesis and curli fiber production, driving the transition to the rdar (red, dry, and rough) morphotype, a surface‐adapted phenotype associated with enhanced persistence on stainless steel and other food contact materials (Pang et al. 2023; Solano et al. 2002). Independent of flagellar sensing, Type 1 fimbriae encoded by the fim gene cluster contribute to surface recognition on hydrophobic and protein‐conditioned interfaces that resist flagellar engagement (Schumann‐Muck et al. 2023). This redundancy in mechanosensory inputs, converging on the same c‐di‐GMP node, underscores why strategies targeting a single adhesion pathway are insufficient for sustained biofilm suppression (Hassanin et al. 2025).

#### 5.1.1 Gram‐Positive Surface Sensing: Structural Distinctions and Stressosome‐Mediated Responses

Gram‐positive pathogens of food safety relevance, notably *L. monocytogenes*, employ surface‐sensing architectures that differ structurally from the flagellar stator mechanism described above, a distinction with direct implications for evaluating the broad‐spectrum utility of plasma‐engineered surfaces. Unlike Gram‐negative bacteria, whose outer membrane lipopolysaccharides mediate initial electrostatic interactions with antimicrobial surfaces, Gram‐positive organisms are bounded by a thick peptidoglycan layer of 20–80 nm that imposes greater steric resistance to ion penetration and reactive species diffusion (Bland et al. 2022).

Initial abiotic surface contact in *L. monocytogenes* is mediated by wall teichoic acids (WTAs) and lipoteichoic acids (LTAs), anionic polymers anchored within the peptidoglycan layer that govern surface charge and early adhesion dynamics (Janež et al. 2021). The interaction of these polymers with plasma‐engineered nanotopographies subjects the rigid cell wall to spatial constraints, generating mechanical stress at the envelope level. This stress is transduced intracellularly through the LisRK two‐component system, a primary surface‐responsive regulator in *L. monocytogenes* that controls adhesion and stress tolerance programs independently of c‐di‐GMP accumulation (Alejandro‐Navarreto and Freitag 2024; Meireles et al. 2024)

A mechanistically distinct feature of Gram‐positive surface sensing is the stressosome, a supramolecular sensory complex conserved across Firmicutes. Mechanical distortion of the cell wall activates the RsbR–RsbS–RsbT complex, initiating a phosphorylation cascade that ultimately activates the alternative sigma factor SigB (Dessaux et al. 2021). SigB functions as the master regulator of the general stress response and biofilm commitment in *L. monocytogenes*, providing a functional parallel to the RpoS–CsgD regulatory axis in *Salmonella* (Bland et al. 2022; Guerreiro et al. 2020).

A further distinction relevant to food processing environments concerns flagellar mechanosensing in *L. monocytogenes*. At host infection temperatures of 37°C, flagellar expression is repressed. In the temperature ranges characteristic of food processing facilities, typically below 30°C, however, *L. monocytogenes* expresses flagella and relies on flagellar motility for initial surface attachment and biofilm initiation (Lemon et al. 2007). This temperature‐dependent regulation indicates that nanotopographical interference with flagellar anchoring retains mechanosensory relevance for *L. monocytogenes* under food industry conditions, contrary to assumptions derived from clinical infection models.

Collectively, these distinctions, summarized in Figure 3, indicate that plasma‐engineered surfaces engage both Gram‐negative and Gram‐positive surface‐sensing networks through complementary mechanisms. Nanotopographical features may impose mechanical constraints on the flagellar stator complex in *Salmonella* and on the WTA/LTA‐peptidoglycan interface in *L. monocytogenes*, while localized generation of ROS/RONS and metal ion diffusion may interfere with downstream regulators, including the *σ* ^B^ adaptation network (Bland et al. 2022; Meireles et al. 2024).

**FIGURE 3:**
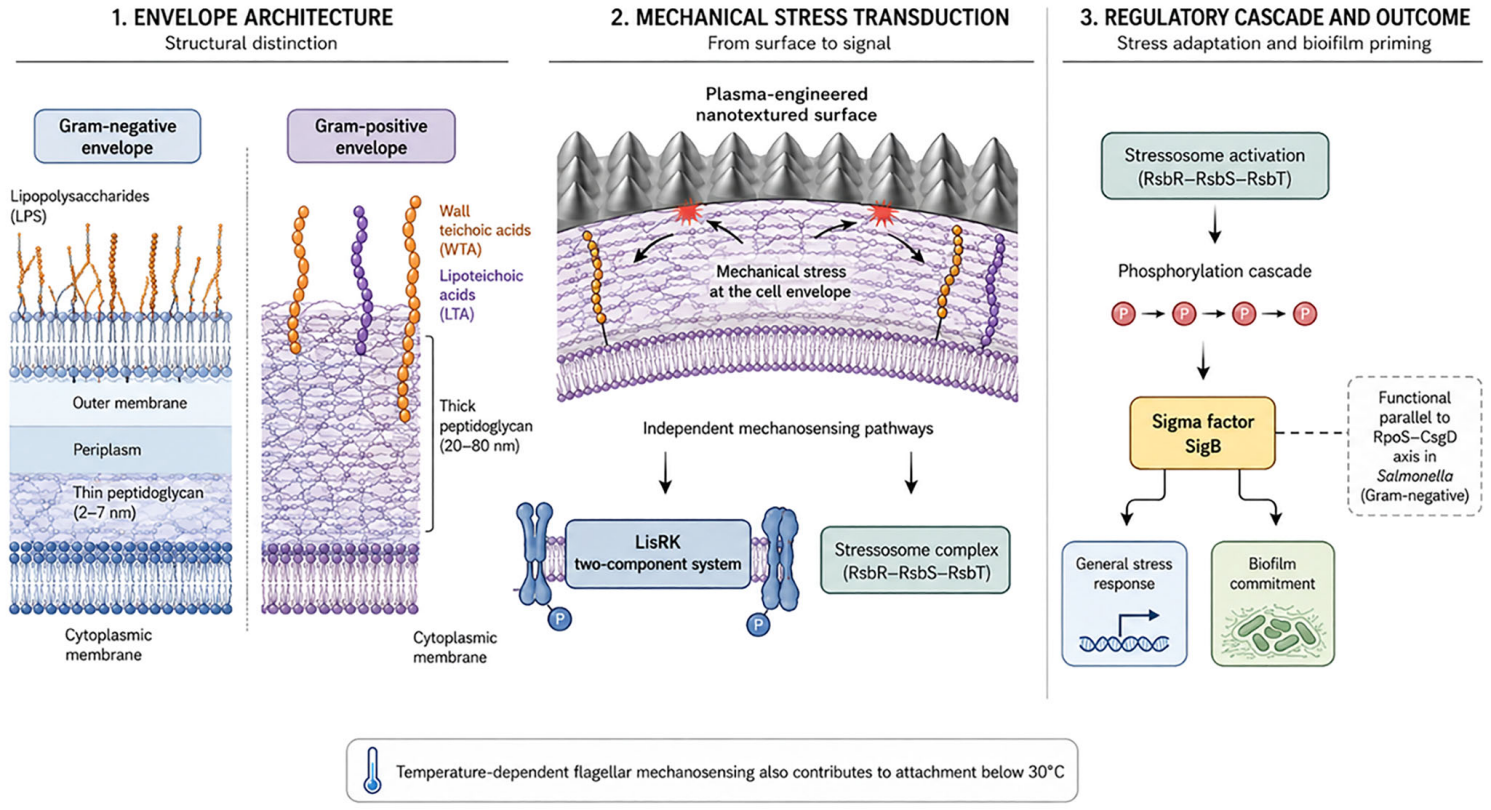
Structural and regulatory distinctions in Gram‐positive surface sensing. Unlike the flagellar mechanotransduction pathway described for *Salmonella*, *Listeria monocytogenes* senses engineered nanotopography through a thick peptidoglycan envelope embedded with wall teichoic acids (WTA) and lipoteichoic acids (LTA) (Bland et al. 2022; Janež et al. 2021). Mechanical stress imposed by nanoscale surface features is transduced independently through two parallel systems: the LisRK two‐component system (Alejandro‐Navarreto and Freitag 2024; Meireles et al. 2024) and the stressosome complex (RsbR–RsbS–RsbT), the latter culminating in activation of the alternative sigma factor SigB, which governs the general stress response and biofilm commitment (Dessaux et al. 2021; Oliveira et al. 2022; Williams et al. 2019). This regulatory axis is functionally analogous to but molecularly distinct from the RpoS–CsgD pathway in *Salmonella* spp. (Bland et al. 2022; Guerreiro et al. 2020). Figure created by the authors based on the mechanisms discussed in Section 5.1 of this review.

#### 5.1.2 Plasma Nanotopography as a Mechanosensory Disruptor

Plasma surface engineering enables precise modulation of surface nanotopography at length scales comparable to bacterial appendages and envelope structures involved in early surface sensing (Lee et al. 2024). Unlike conventional antimicrobial approaches that primarily aim to induce cellular damage after attachment, engineered topographies may interfere with the physical signals required for bacterial commitment to surface colonization. In Gram‐negative pathogens, mechanical constraints imposed on flagellar structures can alter stator loading dynamics, potentially affecting the mechanotransductive processes associated with c‐di‐GMP accumulation and biofilm initiation (Mu et al. 2023). In Gram‐positive organisms, where surface perception relies on a rigid peptidoglycan envelope and associated sensory systems, nanotopographical interactions may instead generate envelope stress that influences regulatory pathways involved in adaptation and persistence (Bland et al. 2022; Meireles et al. 2024).

The relevance of this mechanism extends beyond a single bacterial structure, as foodborne pathogens possess multiple and partially redundant strategies for interpreting surface contact. For *Salmonella* spp., flagellar mechanosensing converges with regulatory networks controlling motility, extracellular matrix production and the transition toward a sessile phenotype. In contrast, *L. monocytogenes* integrates envelope‐derived signals through systems such as LisRK and the stressosome, which regulate *σ* ^B^‐dependent stress adaptation and biofilm‐associated responses. The effectiveness of plasma‐engineered surfaces may depend not only on the magnitude of mechanical perturbation but also on how these physical cues are interpreted by species‐specific regulatory networks.

A critical challenge for the development of mechanosensory‐based antimicrobial surfaces is the translation of nanoscale interactions observed under controlled conditions into complex industrial environments. Bacteria exposed to food processing surfaces encounter simultaneous chemical, mechanical, and nutritional signals derived from conditioning films and fluctuating processing conditions. Thus, future advances will require integrating nano‐topographical design with chemical functionality and biological validation under realistic food matrix conditions. Such approaches may enable surfaces that interfere with early bacterial adaptation while maintaining antimicrobial activity during prolonged industrial use.

Despite increasing evidence that bacterial surface sensing can be influenced by engineered interfaces, a major limitation remains the incomplete understanding of how nanoscale physical cues are translated into species‐specific regulatory outcomes under industrial conditions. Most mechanistic studies rely on simplified surfaces and single‐species models, whereas food processing environments involve conditioning films, mixed microbial communities, and fluctuating physicochemical conditions. Future advances will require coupling surface characterization with quantitative measurements of bacterial mechanotransduction under realistic processing scenarios.

### 5.2 Genetic Regulatory Collapse: Targeting the RpoS–CsgD Axis

Following stable surface attachment, bacteria activate a hierarchical genetic program that consolidates adhesion and promotes biofilm maturation (Sauer et al. 2022). Central to this process is the alternative sigma factor RpoS, which governs the general stress response and regulates the expression of the master biofilm regulator CsgD (Zilelidou et al. 2020). In *Salmonella*, CsgD controls transcription of the *csgBAC* operon encoding curli fimbriae and the *adrA* pathway responsible for cellulose biosynthesis, both critical components of the extracellular polymeric substance matrix (Pang et al. 2023).

Persistent industrial isolates frequently display the rdar morphotype, characterized by elevated curli and cellulose production and enhanced tolerance to desiccation, nutrient limitation and chemical sanitation (Zhai et al. 2024). Once this regulatory state is established, biofilms exhibit high recalcitrance to conventional control measures (Steenackers et al. 2012).

In contrast to diffuse chemical sanitization, which often imposes sublethal stress and promotes adaptive responses, plasma‐engineered surfaces enable localized molecular interference through immobilized antimicrobial ions such as Zn^2+^ and Ag^+^ embedded within nanometric surface matrices (R. Sharma et al. 2025). This regulatory interference precedes overt lethality and directly compromises the genetic commitment to biofilm maturation (J. Wang et al. 2024).

Zn^2+^ and Ag^+^ display strong affinity for thiol groups in enzymes, transcriptional regulators and components of the electron transport chain, disrupting protein conformation, impairing respiratory metabolism and promoting intracellular accumulation of reactive oxygen species (ROS) (X. He et al. 2017). This oxidative burden forces metabolic reallocation toward survival rather than multicellular behaviors (Hadinoto et al. 2023).

Multiple studies indicate that zinc‐ and silver‐based nanostructures suppress biofilm‐associated gene expression in *Salmonella*, including downregulation of *csgD*, *csgA*, and *bcsA* transcription, thereby indirectly reducing curli fimbriae and cellulose production downstream of the RpoS–CsgD axis (Abdelghafar et al. 2022; Dias de Emery et al. 2023). Mechanistically, Zn^2+^ ions disrupt thiol‐dependent transcriptional regulators and generate intracellular oxidative stress, creating metabolic conditions that are incompatible with sustained biofilm commitment (Fulindi et al. 2026; Wei et al. 2022). As a result, even when surface contact occurs, the genetic hierarchy required for rdar morphotype expression fails to consolidate (Steenackers et al. 2012). By targeting the RpoS–CsgD regulatory axis at the ionic level, plasma‐embedded metallic reservoirs collapse biofilm architecture at their genetic foundation, complementing the mechanosensory disruption described in Section 5.1. Importantly, this ionic interference must be distinguished from sublethal exposure: insufficient local concentrations of Zn^2+^ have been shown to select for adaptive resistance in *S*. Typhimurium through *phoQ* mutations that remodel outer membrane composition and paradoxically enhance biofilm formation (Wei et al. 2022), a risk that plasma‐controlled release kinetics aim to mitigate.

The ionic interference described above, however, represents a downstream consequence of surface contact. An upstream regulatory connection, less frequently acknowledged in the surface engineering literature, links specific plasma‐induced surface physicochemical properties directly to bacterial gene expression before ion release (Y. Cheng et al. 2019). Superhydrophobic nanotopographies generated by plasma treatment reduce the effective contact area between the bacterial envelope and the substrate, imposing abnormal mechanical boundary conditions on the cell surface (Mu et al. 2023; Pan et al. 2019).

This geometric mismatch generates periplasmic stress detectable by the Cpx two‐component system, in which membrane‐anchored CpxA undergoes conformational activation and phosphorylates the response regulator CpxR (Price and Raivio 2009). Activated CpxR represses *csgD* transcription and downregulates flagellar biosynthesis genes, effectively preempting the surface commitment cascade at the regulatory level before ionic stress accumulates (Dorel et al. 2006; Steenackers et al. 2012). In parallel, surfaces that impose mechanical perturbation on the peptidoglycan layer activate Rcs phosphorelay through the outer membrane lipoprotein RcsF, with RcsB subsequently modulating the expression of biofilm matrix components and motility regulators (Clarke 2010).

Both systems respond to surface physicochemical properties at length scales directly achievable by plasma‐assisted deposition, establishing a mechanistic continuum from engineered surface properties to specific transcriptional outcomes.

However, the extent to which these regulatory effects persist after exposure to complex food matrices remains insufficiently characterized. Conditioning films may attenuate mechanical interactions and modify ion transport, potentially altering the relationship between engineered surface properties and intracellular responses. Therefore, future studies should integrate transcriptomic approaches with realistic organic fouling models to determine whether regulatory suppression observed under laboratory conditions is maintained during industrial operation.

### 5.3 Preventing VBNC Induction at the Engineered Interface

A well‐documented limitation of conventional industrial sanitation is the induction of the VBNC state (Arvaniti et al. 2021). Exposure to sublethal concentrations of chemical sanitizers, including quaternary ammonium compounds, chlorine derivatives and peracetic acid, has been repeatedly shown to drive foodborne pathogens into dormant yet potentially virulent conditions (Obłąk et al. 2021; Y. Zhao et al. 2023). These cells evade routine culture‐based detection and constitute hidden reservoirs for post‐sanitation recontamination (Fox et al. 2021).

This risk is not restricted to chemical disinfectants. Atmospheric plasma treatments applied at insufficient doses can also induce VBNC phenotypes, particularly within biofilms, when the imposed oxidative stress remains below lethal thresholds (Y. M. Zhao, Patange, et al. 2020). These observations underscore a fundamental principle, microbial fate is dictated not solely by the nature of the antimicrobial agent, but also by the spatial distribution and energetic intensity of the applied stress (S. Sharma et al. 2022).

Plasma‐engineered surfaces may address this limitation by concentrating antimicrobial activity directly at the solid–liquid interface, where bacterial contact occurs (Hage et al. 2022). Unlike transient plasma jets or bulk liquid sanitizers that dissipate rapidly and form sublethal concentration gradients, plasma‐deposited coatings integrate antimicrobial functionality into the surface itself (Dufour 2023). Bacteria encountering these interfaces may be exposed to localized ionic and oxidative stress levels sufficient to overwhelm cellular repair mechanisms under controlled conditions (Middlemiss et al. 2023).

In addition to metabolic disruption, plasma‐engineered surfaces induce irreversible physical damage, including membrane permeabilization, envelope destabilization and oxidative DNA injury (Scholtz et al.^2021^). These effects differ fundamentally from the reversible stress responses elicited by sublethal chemical exposure and substantially reduce the likelihood of recovery or entry into dormancy (Obłąk et al. 2021).

By combining hydration control, localized oxidative stress and controlled ionic activity, plasma‐engineered interfaces mitigate the chemical gray zone associated with VBNC induction (Y. Yan et al. 2026). In contrast to sublethal chemical exposure, which may promote gradual stress adaptation, plasma‐engineered interfaces may shift bacterial outcomes toward effective surface repulsion or irreversible cellular damage, potentially reducing the emergence of dormant subpopulations under conditions of adequate local stress intensity (Bharathi et al. 2025).

Nevertheless, preventing VBNC induction requires careful control of antimicrobial intensity, as insufficient stress levels may favor adaptation rather than elimination. Determining the threshold between lethal and adaptive responses under dynamic food‐processing conditions remains a critical challenge for plasma‐engineered systems.

### 5.4 Multilayered Molecular Suppression of Persistence

Plasma surface engineering suppresses bacterial persistence through an integrated molecular strategy targeting perception, regulation and survival. First, plasma‐induced nanotopographies generate mechanosensory instability that disrupts surface recognition and inhibits c‐di‐GMP‐mediated commitment to sessile lifestyles (Linklater et al. 2021; F. Song et al. 2018).

Second, embedded antimicrobial ions and surface‐bound reactive species interfere with global stress regulators governing biofilm development. In Gram‐negative foodborne pathogens, this interference converges on the RpoS–CsgD axis, whereas in Gram‐positive organisms, it targets the homologous stress response controlled by the alternative sigma factor SigB (Bland et al. 2022). Disruption of these master regulators suppresses coordinated expression of extracellular matrix components, including curli, cellulose, eDNA, and associated adhesins (Flemming et al. 2025).

Third, localized generation of RONS at the engineered interface enforces lethal stress upon bacterial contact (Y. Zhao et al. 2023). Coupled with physical membrane damage, this localized assault overwhelms repair mechanisms and minimizes entry into the VBNC state frequently induced by sublethal sanitation (Arvaniti et al. 2021).

This multilayered framework offers a mechanistic basis for the improved performance reported for plasma‐engineered surfaces relative to conventional sanitation and static antifouling coatings under controlled experimental conditions (Chowdhury et al. 2025). By simultaneously targeting bacterial sensing, genetic regulation, and viability, these interfaces may substantially constrain the adaptive responses available to foodborne pathogens (Katsigiannis et al. 2022).

This molecular perspective completes the conceptual link between interfacial physics and microbial biology. Plasma‐engineered surfaces may exert a coordinated, multilayer influence extending beyond initial adhesion, encompassing mechanosensing disruption, regulatory interference, and suppression of persistence‐associated stress responses, although direct validation under food‐relevant organic loads remains a priority research gap. This integrated framework provides the foundation for the final section of this review, which addresses translational challenges, regulatory considerations and future directions for plasma‐based antimicrobial surfaces in food processing environments.

While Figure 4 illustrates the integrated suppression cascade specific to plasma‐engineered interfaces, Table 5 extends this analysis to a comparative framework, contextualizing plasma‐based strategies relative to conventional and emerging alternatives with respect to molecular targets, persistence pathway interference, VBNC induction risk, and operational stability under food processing demands.

**FIGURE 4:**
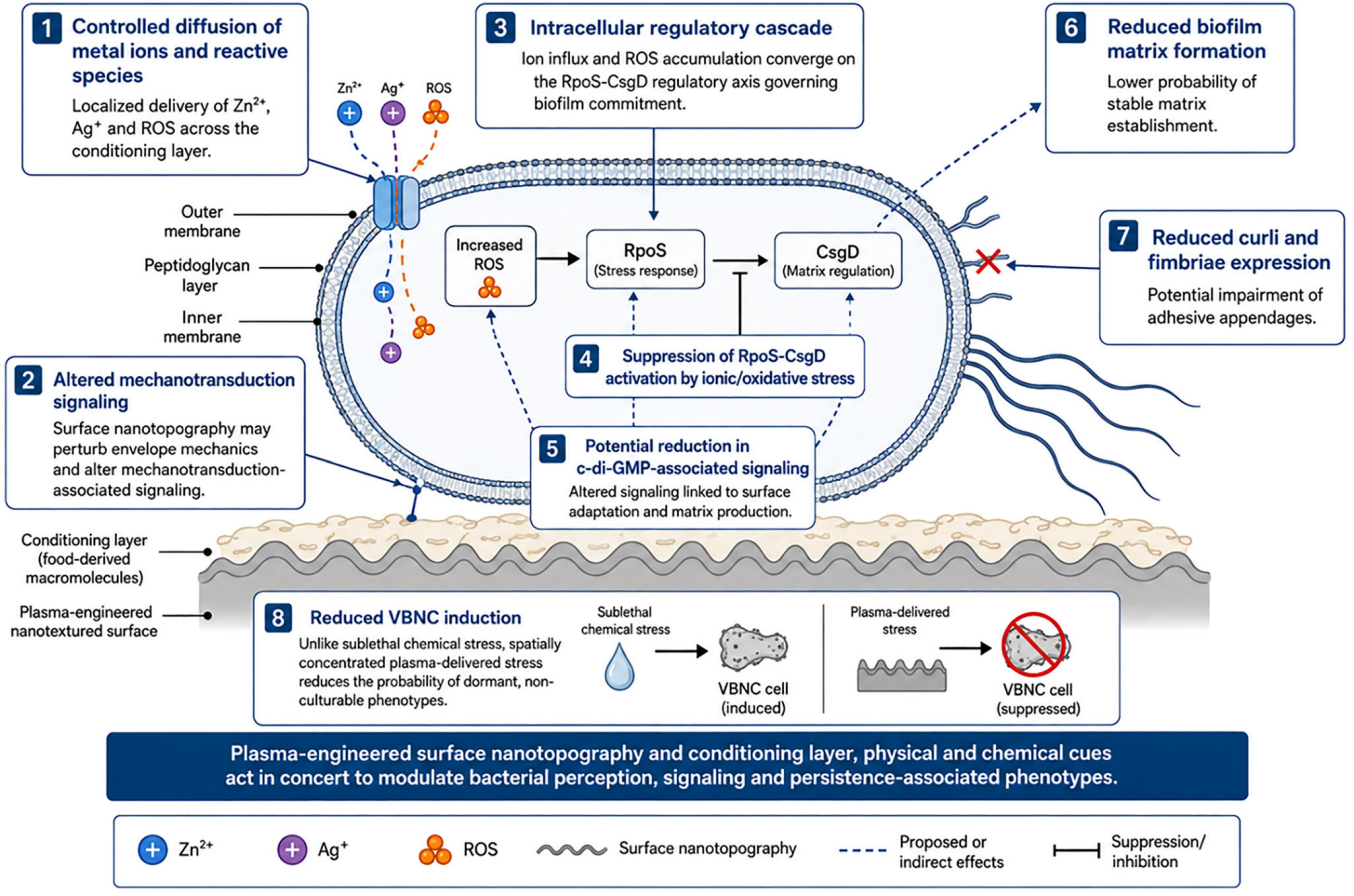
Integrated molecular suppression of *Salmonella* persistence on plasma‐engineered food contact surfaces. Surface nanotopography perturbs envelope mechanics and disrupts mechanotransduction, interfering with early signaling pathways associated with biofilm initiation (Linklater et al. 2021; F. Song et al. 2018). In parallel, controlled diffusion of metal ions and interfacial reactive species imposes localized oxidative and ionic stress, modulating global regulatory networks linked to extracellular matrix synthesis (Bland et al. 2022; Flemming et al. 2025). The convergence of mechanical and chemical stress at the interface limits curli and fimbriae expression, constrains matrix assembly, and reduces the probability of persistence‐associated phenotypes, including the transition toward viable but non‐culturable states (Y. Zhao et al. 2023; Arvaniti et al. 2021). Dashed arrows denote simplified, bidirectional c‐di‐GMP signaling. Figure created by the authors based on the mechanisms discussed in Section 5.4 of this review.

**TABLE 5:** Molecular targets and detection methods reported for surface control strategies against *Salmonella* and related foodborne pathogens.

Strategy	Substrate/pathogen	Molecular target	Detection method	Quantified outcome	References
Chemical sanitizer	Stainless steel and polystyrene/*Salmonella* Typhimurium, *Salmonella* Heidelberg	*csgD*, *csgA*, *csgB*, *bcsE*, *envZ*, *spiA*, *ycfR*	RT‐qPCR	Rugose vs. smooth morphotype: *envZ* 139.5‐fold, *csgD* 100.3‐fold, *csgB* 11.0‐fold, *csgA* 2.84‐fold, *bcsE* 2.35‐fold (*p* ≤ 0.05)	Bansal et al. (2019)
Chemical sanitizer^a^	Planktonic cells/*Salmonella* Enteritidis	*csgD*, *agfA*, *adrA*, *bapA*, *luxS*, *sdiA*	RT‐qPCR	All targets upregulated more than twofold under sublethal chlorine stress relative to non‐stressed cells	Zarei et al. (2023)
Chemical sanitizer	Stainless steel and plastic/*S*. Enteritidis	*hilA*, *hilC*, *flhD*, *csgA*, *csgD*, *sdiA*, *rpoS*	RT‐qPCR	0.01% *trans*‐cinnamaldehyde nanoemulsion, 24 h at 25°C: relative fold change 0.27 (*hilA*), 0.29 (*hilC*), 0.37 (*flhD*), 0.27 (*csgA*), 0.35 (*csgD*), 0.28 (*sdiA*), all *p* < 0.05; *rpoS* 0.68, not significant	Shah et al. (2025)
Chemical sanitizer	Polystyrene/*S*. Enteritidis	*luxS*, *csgD*, *adrA*, *csgB*, *sdiA*	RT‐qPCR	Subinhibitory ethanol: *luxS* upregulated 2.49‐ to 10.08‐fold; *csgD*, *adrA*, *csgB*, and *sdiA* unchanged	S. He et al. (2022)
Chemical sanitizer^a^	Suspension/*S*. Enteritidis	*emrB*, *kdpABC*, TCA cycle genes	RNA‐seq, RT‐qPCR	Sodium hypochlorite stress upregulated efflux systems and downregulated potassium transport and TCA cycle genes, associated with VBNC entry	S. Wang et al. (2022)
Chemical sanitizer^a^	Liquid medium, non‐stressed exponential phase/*S*. Enteritidis	CpxR (Δ*cpxR*), *ompC*, *ompN*	Quantitative proteomics, gene knockout	Δ*cpxR* vs. wild‐type: *ompC* fold change 0.50 (*p* = 0.002), *ompN* fold change 0.35 (*p* = 0.019)	X. Liu et al. (2021)
Plasma‐activated water	Stainless steel and polystyrene/*L. monocytogenes*	*cbiD*, *cbiH*, *eutBCL*, *accB*, *fabI*, *actA*, *hly*, *bcrB*	RNA‐seq	Sessile cells: *cbiH* log_2_FC 6.37, *bcrB* multidrug efflux induced; virulence operons (*actA*, *hly*, *plcB*) repressed	Fernández‐Gómez et al. (2023)
Plasma‐activated water	Stainless steel/*Escherichia coli* biofilm	*trxC*, *cysP*, *nuoM*, *ytfE*	RNA‐seq, gene knockout	Sublethal exposure: *trxC* 4.23‐fold, *cysP* 1.58‐fold, *nuoM* 1.74‐fold upregulated; Δ*trxC*, Δ*cysP*, and Δ*nuoM* mutants showed reduced biofilm viability and intracellular RONS accumulation	Vyas et al. (2025)
Plasma‐activated water^a^	Suspension/*Salmonella* Newport	*pagC*, *sodA*, *katE*, *trxA*, *ompA*, *ompC*, *ompD*, *ompF*	RT‐qPCR, Western blot	*pagC* upregulated 4.66‐ to 13.52‐fold, *trxA* 6.02‐fold; intracellular ATP reduced 38%–65%, with VBNC induction and retained virulence determinants	Sun et al. (2024)
Plasma‐activated water^a^	Suspension/*S*. Enteritidis	*ompA*, *secA*, TCA cycle, PTS transporters	RNA‐seq	223 differentially expressed genes (96 upregulated, 127 downregulated); *secA* repression associated with impaired protein translocation	Qian et al. (2024)
Plasma‐activated water^a^	Sequential sublethal exposure/*S*. Typhimurium	*qseC*, *mreB*, *cadBC*, *arcB*, *zntB*, *csrA*, *rpoD*, *rpoA*	Whole‐genome sequencing	Non‐synonymous mutations fixed in four resistant variants, conferring cross‐tolerance to sodium hypochlorite, peracetic acid, and carvacrol	Pagán et al. (2024)

*Note*: Entries are restricted to primary studies reporting experimental measurement of gene or pathway expression (RT‐qPCR, RNA‐seq, quantitative proteomics, gene knockout, reporter fusion, or EMSA); studies reporting only phenotypic endpoints (log reduction, biofilm biomass, microscopy) are compiled in Table 4.

Abbreviations: DEG, differentially expressed gene; EMSA, electrophoretic mobility shift assay; PTS, phosphotransferase system; RONS, reactive oxygen and nitrogen species; TCA, tricarboxylic acid; VBNC, viable but non‐culturable.

^a^ Mechanisms characterized in planktonic suspension rather than on an abiotic contact surface, cited to support the regulatory pathway rather than surface performance. Plasma‐activated water is listed separately from plasma‐engineered surfaces, as it constitutes a liquid sanitizing treatment rather than a permanently modified interface. A systematic search across Scopus and PubMed, complemented by reference back‐tracing of published reviews on antimicrobial and antifouling coatings, identified no primary study reporting molecular‐level characterization of persistence‐associated regulators under contact‐killing coatings, zwitterionic or PEG‐based antifouling coatings, or plasma‐deposited functional films; for these strategies, the available evidence remains restricted to adhesion and log‐reduction endpoints.

### 5.5 Translational Validation Gaps and Industrial Constraints

Despite substantial recent growth in the antimicrobial surface literature, important discrepancies persist between bench‐scale validation conditions and the operational demands of food‐processing environments. Several consistent gaps emerge when this evidence is assessed against industrial applicability criteria.

#### 5.5.1 Absence of CIP‐Cycle Validation

A recurring methodological feature across studies is the assessment of ion release and surface stability in simplified aqueous systems, typically phosphate‐buffered saline or deionized water, over relatively short timeframes and without chemical or mechanical stress. Yassin et al. (2019) quantified Ag^+^ release from polydopamine‐mediated coatings in PBS at 37°C over 7 days, reporting sustained concentrations between 2 and 4 µg/mL. Coughlan et al. (2008) evaluated Zn^2+^ and Ag^+^ elution from glass polyalkenoate cements in purified water for up to 30 days. Valappil and Higham (2014) monitored biofilm inhibition and ion release in aqueous systems for up to 72 h. None of these investigations simulated repeated industrial CIP cycles combining elevated temperature, extreme pH, and hydrodynamic shear.

Industrial CIP conditions are not simply intensified laboratory assays; they involve combined physicochemical stresses that may alter coating integrity, ion release kinetics, and long‐term surface functionality (Gavahian et al. 2024). In the absence of such validation, extrapolation to continuous industrial operation remains uncertain.

#### 5.5.2 Absence of Longitudinal Industrial Monitoring

Surface performance was not monitored beyond short experimental periods in the analyzed literature. Kroll et al. (2016) conducted 18‐day continuous exposure experiments in artificial stream systems, focusing on benthic microbial communities rather than food‐contact surfaces. Coughlan et al. (2008) extended elution studies to 30 days under static immersion, while studies in the biomedical implant literature have followed biofilm reduction for periods of up to 2 weeks posttreatment.

Long‐term parameters relevant to food processing, including progressive coating degradation, hydrophobic recovery, loss of antimicrobial efficacy, and mechanical stability under repeated use, were not evaluated. Consequently, durability estimates under industrial conditions remain largely inferential.

#### 5.5.3 Absence of Mass Transport Modeling Through Organic Matrices

Although several studies have quantified ionic concentrations in the surrounding aqueous phase, none have characterized ion transport within mature biofilms. Available studies report bulk ion concentrations but do not determine diffusion coefficients, spatial concentration gradients, or penetration depth across structured extracellular polymeric substance matrices. Recent evidence indicates that spatial heterogeneity within EPS generates anomalous diffusion patterns that immobilize antimicrobial particles before reaching bacterial cells (Coppens et al. 2023; Lu et al. 2024).

Moreover, all evaluated systems were aqueous or environmentally simulated. Ion diffusion through food‐derived conditioning films composed of proteins, lipids, and polysaccharides adhered to stainless steel or plasma‐modified substrates has not been quantified. Predictive models of metal bioavailability at the biofilm–surface interface under industrial organic fouling conditions are therefore lacking.

#### 5.5.4 Adaptive Tolerance Under Chronic Sublethal Exposure

Chronic sublethal exposure to metals may theoretically promote adaptive responses, as supported by broader microbiological literature. Within the studies analyzed here, however, direct experimental evidence remains limited. Valappil and Higham (2014) reported partial biofilm re‐emergence after 48–72 h, proposing possible diffusion barriers or efflux‐related mechanisms. Kroll et al. (2016) observed shifts in benthic microbial community composition following 18 days of sublethal silver exposure, suggesting ecological adaptation dynamics without mechanistic resolution. More recently, evidence from food processing environments indicates that sublethal antimicrobial exposure selects for increased minimum inhibitory concentrations in *Salmonella*, with tolerance emerging after repeated cycles of exposure (Obe et al. 2024).

The literature consistently demonstrates short‐term antimicrobial efficacy. However, the parameters that determine whether such efficacy is maintained under real industrial conditions, including resistance to repeated CIP cycles, functional aging during continuous operation, ion transport through complex organic matrices, and chronic adaptive responses, remain insufficiently characterized. Addressing these aspects through longitudinal industrial studies, mechanistic transport modeling, and evolutionary microbiology approaches represents a necessary research agenda to support the reliable implementation of plasma‐functionalized antimicrobial surfaces in food‐processing systems.

A further underexplored dimension concerns multispecies biofilm dynamics. Industrial food contact surfaces are rarely colonized by single‐species populations; instead, structured consortia emerge in which interspecies metabolic interactions, matrix‐mediated shielding, and cooperative stress tolerance may substantially attenuate antimicrobial efficacy beyond what single‐species models predict (Flemming et al. 2025; J. Yan and Bassler 2019). Whether plasma‐engineered surfaces retain mechanosensory and ionic efficacy against multispecies biofilms under food‐relevant organic loads remains uncharacterized and constitutes an important validation gap.

#### 5.5.5 Mechanical Degradation and Biofilm‐Permissive Surface Damage

A concern that receives limited attention in the antimicrobial surface literature is the mechanical durability of plasma‐deposited films under the operational demands of food processing facilities. Industrial hygienization routinely involves daily mechanical scrubbing, high‐pressure water jets, and repeated exposure to corrosive alkaline and acidic CIP agents at elevated temperatures. Under these conditions, thin‐film coatings, including those produced by PECVD and magnetron sputtering, are susceptible to progressive delamination, microcracking, and localized abrasion (van den Berg et al. 2024).

The consequences of partial film degradation extend beyond simple loss of antimicrobial function. Surface defects such as scratches, delamination edges, and microcracks create geometric discontinuities that increase the effective surface area, trap organic residues, and generate sheltered microenvironments with reduced sanitizer accessibility. These physical niches are well‐established as preferential sites for biofilm initiation, as they provide mechanical protection from shear forces and limit exposure to cleaning agents (Whitehead et al. 2019). Whether plasma‐deposited films, upon degradation, generate surface architecture that actively promotes rather than suppresses biofilm formation remains an open and practically important question. To date, no systematic study has evaluated the biofilm‐permissive consequences of plasma film failure under industrially relevant mechanical stress cycles. This gap remains uncharacterized and should be addressed before large‐scale deployment.

#### 5.5.6 Scalability Constraints and Atmospheric Plasma Alternatives

The deposition techniques most frequently employed to produce nanostructured antimicrobial coatings, including PECVD and magnetron sputtering, are inherently vacuum‐dependent processes. While these methods offer precise control over film stoichiometry, thickness, and nanostructural architecture, their operational requirements impose significant constraints on industrial scalability. Treatment of large, geometrically complex food‐contact surfaces, such as conveyor belts spanning tens of meters, internal pipe networks, or irregular‐profile cutting equipment, is technically challenging and economically prohibitive under vacuum‐chamber configurations, given the physical constraints imposed by chamber size and line‐of‐sight deposition requirements inherent to these techniques.

APP systems represent a technically promising alternative in this context. Operating without vacuum infrastructure, APP jets and DBD devices can be deployed in open industrial environments, adapted to continuous processing lines, and applied to surfaces of arbitrary geometry (Linklater et al. 2021). Where the antimicrobial functionalities described in this review, including metal ion embedding, nanotopography generation, and surface energy modulation, can be replicated under atmospheric conditions, APP‐based deposition would substantially reduce the translational barrier between laboratory validation and industrial implementation. Current evidence suggests that several of these functional outcomes are achievable via APP, although systematic comparative data against vacuum‐deposited equivalents under food‐relevant conditions remain limited and constitute an important direction for future research.

### 5.6 Future Perspectives

Although recent advances in plasma deposition technologies have substantially improved coating stability, adhesion and antimicrobial performance under laboratory conditions, translation to industrial food processing remains at an early stage, with limited validation under realistic operating conditions.

Translating plasma‐engineered food contact surfaces from laboratory proof‐of‐concept to industrial implementation requires confronting limitations that remain insufficiently investigated in the current literature. Chief among these is the absence of systematic data on long‐term coating behavior under the cumulative mechanical and chemical stress of real processing environments. Near‐term progress depends on adopting multilayer deposition architectures, combining silicon oxycarbide or DLC interlayers with HiPIMS, which increases film density and adhesion while suppressing the columnar void formation that renders conventional sputtered coatings vulnerable to hydrolytic infiltration under CIP conditions (Sarakinos et al. 2010; Schlebrowski et al. 2021). Whether these architectural improvements are sufficient to sustain antimicrobial functionality across the full operational life of food processing equipment remains an open question that bench‐scale durability studies cannot answer.

A related challenge concerns the temporal stability of plasma‐modified surfaces in contact with food matrices. Progressive hydrophobic recovery through segmental chain relaxation, combined with rapid conditioning film deposition, can progressively attenuate the interfacial properties that plasma engineering is designed to preserve (Dufour 2023; Hua and Zhu 2024a). Post‐plasma stabilization strategies, including zwitterionic polymer grafting and fluorinated PECVD co‐processing to achieve superhydrophobic textures resistant to macromolecule retention, offer near‐term mitigation (Xu et al. 2019; Souza et al. 2020). Longer‐term solutions may require moving beyond static surface design entirely, toward adaptive interfaces capable of responding dynamically to fouling conditions, a direction that remains conceptually promising but experimentally underdeveloped (Linklater et al. 2021; van den Berg et al. 2024).

Scalability presents a structural rather than an incremental challenge. Vacuum‐based deposition processes offer the precision required for functional thin‐film production but are incompatible with the geometric complexity and throughput demands of industrial food processing facilities (Katsigiannis et al. 2022). APP systems, including DBD and plasma jet configurations, represent a more realistic near‐term pathway, particularly for continuous roll‐to‐roll processing and localized modification of high‐risk components (Alaguthevar et al. 2024; Dufour 2023). Extending this capability to three‐dimensional internal surfaces, pipe networks, and assembled equipment without vacuum infrastructure remains a longer‐term engineering objective whose resolution would substantially reduce the translational barrier between laboratory validation and commercial deployment.

Finally, regulatory acceptance is not a peripheral concern but a determinant constraint on the entire translational trajectory. The migration of nanostructures, unreacted precursor oligomers, and metal ions such as Ag^+^ and Cu^2+^ into food matrices will need to be quantified under realistic exposure conditions before FDA and EFSA approval pathways can be meaningfully pursued (Katsigiannis et al. 2022; Mahmoudi‐Qashqay et al. 2023). Standardized migration assays using official food simulants coupled with ICP‐MS detection represent the immediate methodological priority, establishing toxicological baselines for systems such as Ag/a‐C:H nanocomposites under conditions that include both food contact and repeated CIP exposure (Vaidulych et al. 2017). Beyond individual product approval, the field will ultimately require an internationally harmonized regulatory framework specifically designed for plasma‐engineered food contact materials, a structural gap whose resolution depends as much on regulatory science as on materials engineering (Chowdhury et al. 2025; Katsigiannis et al. 2022). These translational requirements, spanning cleaning resistance, mass transport, mechanical durability and regulatory clearance, are synthesized in Figure 5.

**FIGURE 5:**
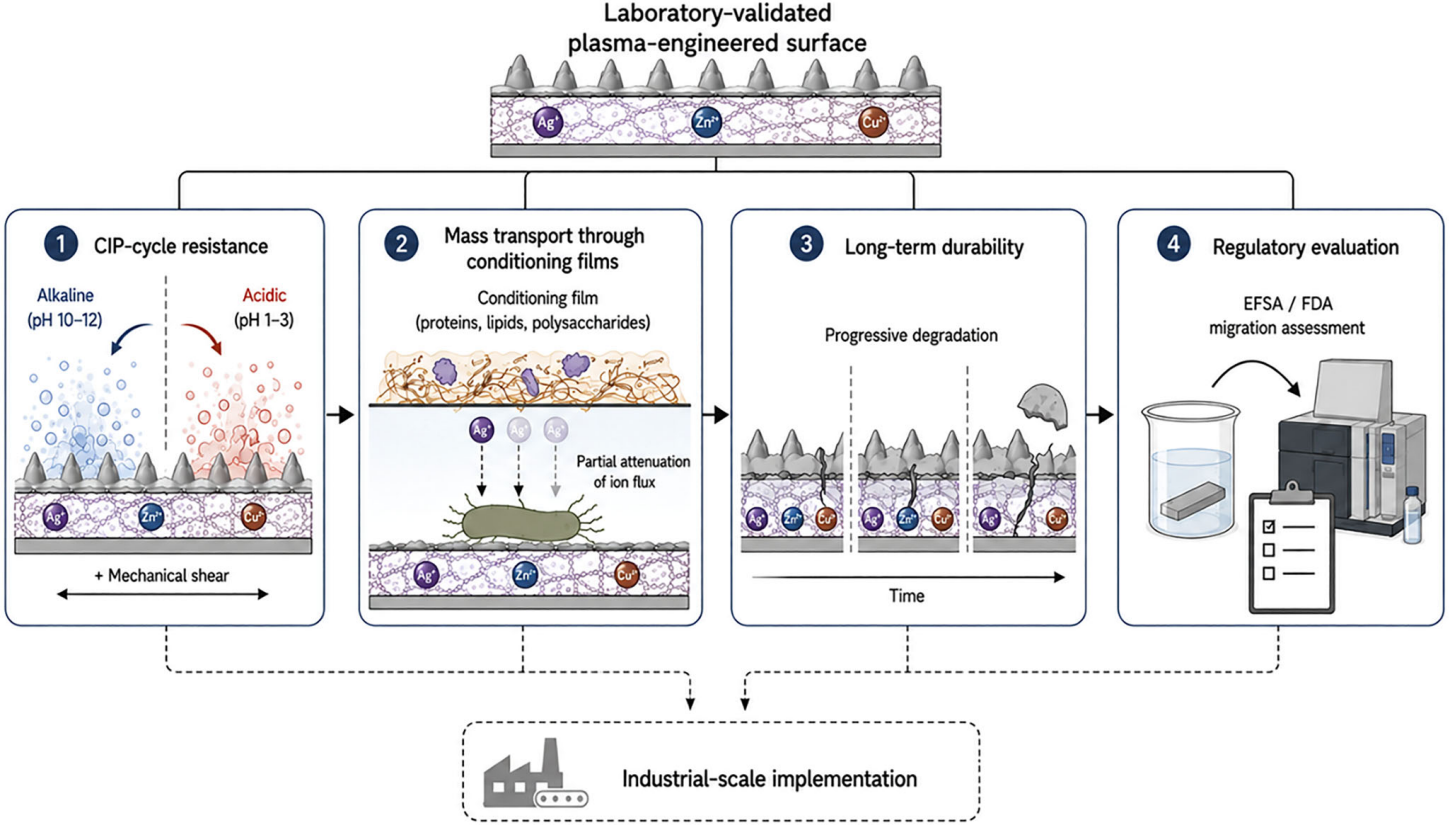
Translational validation requirements for plasma‐engineered antimicrobial surfaces in food processing environments. Laboratory‐validated plasma‐engineered surfaces must pass through four sequential validation domains before industrial deployment: resistance to cleaning‐in‐place (CIP) cycling, mass transport of antimicrobial species through food matrix conditioning films, long‐term mechanical and chemical durability, and regulatory evaluation of ion and nanomaterial migration (e.g., EFSA/FDA assessment). Industrial‐scale implementation (dashed outline) remains a translational target rather than an achieved outcome, reflecting the validation gaps identified throughout Section 5.5. Figure created by the authors based on the validation gaps discussed in Section 5.5 of this review, informed by Yassin et al. (2019), Coughlan et al. (2008), Valappil and Higham (2014), Kroll et al. (2016), Coppens et al. (2023), Gavahian et al. (2024), and Katsigiannis et al. (2022).

## 6 Conclusion

The persistence of foodborne pathogens on food contact surfaces is not solely a consequence of insufficient sanitation intensity but reflects fundamental interfacial constraints imposed by complex food matrices. Immediately after cleaning, residual organic molecules rapidly modify the solid–liquid interface, generating conditioning films that alter surface chemistry, mask engineered functionalities, and create favorable conditions for microbial attachment (Hua and Zhu 2024a). Consequently, antimicrobial surfaces that demonstrate high efficacy under simplified laboratory conditions frequently experience reduced performance when challenged with protein‐rich and chemically complex industrial environments (Katsigiannis et al. 2022; Y. Cheng et al. 2023).

Plasma surface engineering provides a conceptual framework to overcome these limitations by integrating physicochemical surface control with microbial mechanisms of persistence. Rather than relying exclusively on direct bacterial killing, plasma‐functionalized interfaces may combine antifouling properties, controlled antimicrobial ion release, reactive species generation, and interference with bacterial surface sensing pathways (Dufour 2023; Hage et al. 2022). This multilayered strategy expands the role of food contact materials from passive barriers toward active interfaces capable of influencing the early stages of microbial adaptation.

Despite these advances, the translation of plasma‐engineered surfaces into industrial applications remains limited by critical knowledge gaps. The long‐term stability of coatings under repeated CIP cycles, the transport of antimicrobial species through conditioning films and mature biofilms, and the consequences of progressive mechanical degradation remain insufficiently characterized under realistic food‐processing conditions (Yassin et al. 2019; Coughlan et al. 2008; Coppens et al. 2023; van den Berg et al. 2024). Furthermore, determining whether chronic exposure to suboptimal antimicrobial release profiles may promote adaptive tolerance remains essential for evaluating the long‐term microbiological safety of these technologies (Wei et al. 2022; Obe et al. 2024).

Future progress will require a transition from proof‐of‐concept demonstrations toward integrated validation platforms combining materials characterization, microbial physiology, transport modeling, and regulatory assessment. Near‐term advances are expected from improved coating architectures with enhanced adhesion and durability, as well as APP approaches capable of reducing manufacturing constraints and enabling treatment of complex industrial geometries (Sarakinos et al. 2010; Schlebrowski et al. 2021; Linklater et al. 2021). Longer‐term development may involve adaptive interfaces capable of responding dynamically to environmental conditions, including organic loading, moisture variation, and microbial colonization states (Navascués et al. 2025).

Further research priority is the integration of plasma surface engineering with molecular microbiology approaches. Understanding how engineered interfaces influence bacterial mechanosensing, stress adaptation, and persistence‐associated regulatory networks will be essential for designing surfaces that prevent biofilm establishment rather than merely eliminating mature populations (L. Wang et al. 2023). Such strategies should incorporate evolutionary and genomic monitoring frameworks to evaluate the potential emergence of adaptive responses during prolonged exposure (Kroll et al. 2016; L. Wang et al. 2023).

Overall, plasma surface engineering should be viewed as a complementary strategy within integrated food safety systems rather than a replacement for conventional sanitation. By modifying the physicochemical environment encountered by bacteria at the earliest stages of colonization, plasma‐functionalized surfaces offer a promising route toward more durable microbial control. However, their successful implementation will depend on rigorous validation under realistic industrial conditions, standardized evaluation protocols, and coordinated advances in materials science, microbiology, and regulatory science (Mevo et al. 2021).

## Author Contributions

**Rafael Bianchini Fulindi**: conceptualization, investigation, writing – original draft, writing – review and editing, visualization, validation, methodology, formal analysis, supervision, data curation. **Argemiro Soares da Silva Sobrinho**: conceptualization, writing – original draft, validation, visualization, writing – review and editing, project administration, supervision, resources, funding acquisition. **Anderson S. Sant'ana**: conceptualization, investigation, funding acquisition, writing – original draft, writing – review and editing, visualization, project administration, supervision.

## Conflicts of Interest

Rafael Bianchini Fulindi reports a patent (issued and pending) assigned to São Paulo State University (UNESP). The other authors report no conflicts of interest.
